# An Update on Secular Trends in Physical Fitness of Children and Adolescents from 1972 to 2015: A Systematic Review

**DOI:** 10.1007/s40279-020-01373-x

**Published:** 2020-11-07

**Authors:** Thea Fühner, Reinhold Kliegl, Fabian Arntz, Susi Kriemler, Urs Granacher

**Affiliations:** 1 Division of Training and Movement Sciences, Research Focus Cognition Sciences, University of Potsdam, Am Neuen Palais 10, Building 12, 14469 Potsdam, Germany; 2 Epidemiology, Biostatistics and Prevention Institute, University of Zurich, Hirschengraben 84, 8001 Zurich, Switzerland

## Abstract

**Background:**

There is evidence that physical fitness of children and adolescents (particularly cardiorespiratory endurance) has declined globally over the past decades. Ever since the first reports on negative trends in physical fitness, efforts have been undertaken by for instance the World Health Organization (WHO) to promote physical activity and fitness in children and adolescents. Therefore, it is timely to re-analyze the literature to examine whether previous reports on secular declines in physical fitness are still detectable or whether they need to be updated.

**Objectives:**

The objective of this systematic review is to provide an ‘update’ on secular trends in selected components of physical fitness (i.e., cardiorespiratory endurance, relative muscle strength, proxies of muscle power, speed) in children and adolescents aged 6–18 years.

**Data Sources:**

A systematic computerized literature search was conducted in the electronic databases PubMed and Web of Science to locate studies that explicitly reported secular trends in physical fitness of children and adolescents.

**Study Eligibility Criteria:**

Studies were included in this systematic review if they examined secular trends between at least two time points across a minimum of 5 years. In addition, they had to document secular trends in any measure of cardiorespiratory endurance, relative muscle strength, proxies of muscle power or speed in apparently healthy children and adolescents aged 6–18 years.

**Study Appraisal and Synthesis Methods:**

The included studies were coded for the following criteria: nation, physical fitness component (cardiorespiratory endurance, relative muscle strength, proxies of muscle power, speed), chronological age, sex (boys vs. girls), and year of assessment. Scores were standardized (i.e., converted to *z* scores) with sample-weighted means and standard deviations, pooled across sex and year of assessment within cells defined by study, test, and children’s age.

**Results:**

The original search identified 524 hits. In the end, 22 studies met the inclusion criteria for review. The observation period was between 1972 and 2015. Fifteen of the 22 studies used tests for cardiorespiratory endurance, eight for relative muscle strength, eleven for proxies of muscle power, and eight for speed. Measures of cardiorespiratory endurance exhibited a large initial increase and an equally large subsequent decrease, but the decrease appears to have reached a floor for all children between 2010 and 2015. Measures of relative muscle strength showed a general trend towards a small increase. Measures of proxies of muscle power indicated an overall small negative quadratic trend. For measures of speed, a small-to-medium increase was observed in recent years.

**Limitations:**

Biological maturity was not considered in the analysis because biological maturity was not reported in most included studies.

**Conclusions:**

Negative secular trends were particularly found for cardiorespiratory endurance between 1986 and 2010–12, irrespective of sex. Relative muscle strength and speed showed small increases while proxies of muscle power declined. Although the negative trend in cardiorespiratory endurance appears to have reached a floor in recent years, because of its association with markers of health, we recommend further initiatives in PA and fitness promotion for children and adolescents. More specifically, public health efforts should focus on exercise that increases cardiorespiratory endurance to prevent adverse health effects (i.e., overweight and obesity) and muscle strength to lay a foundation for motor skill learning.

**Electronic supplementary material:**

The online version of this article (10.1007/s40279-020-01373-x) contains supplementary material, which is available to authorized users.

## Key Points

**Table:** 

This systematic review documents a large initial increase and an equally large subsequent decrease for cardiorespiratory endurance, but the decrease appears to have reached a floor for all children between 2010 and 2015. Relative muscle strength showed a small increase. Proxies of muscle power indicated an overall small negative quadratic trend. Speed showed small-to-medium increases in recent years.
Because of the different trends in physical fitness, we recommend that already existing programs with the goal to promote PA and fitness should be maintained. More specifically, public health efforts should focus on exercise that increases cardiorespiratory endurance to prevent adverse health effects (overweight, obesity) and muscle strength to lay a foundation for motor skill learning.

## Introduction

The World Health Organization (WHO) recommends at least 60 min of moderate-to-vigorous physical activity (PA) daily and additionally muscle and bone strengthening activities three times per week for children and adolescents aged 5–17 years [1]. Recently published studies showed that a majority of children and adolescents (~ 80%) around the globe do not meet the recommended level of 60 min PA per day [2–4]. Children and adolescents who do not adhere to WHO recommendations [1], they are supposed to suffer from ‘exercise deficit disorder’ including all negative health consequences [5].

Childhood is an important developmental stage to acquire fundamental movement skills through daily PA in order to obtain motor skill competence and movement confidence. Children who do not gain such competencies due to sedentariness are more likely to experience adverse health outcomes later in life [5]. Furthermore, it has been postulated that a physically active lifestyle during childhood and adolescence is robust and tracks into adulthood [6–9]. For instance, Telama et al. [9] conducted a 27-year follow-up measurement of 3,596 Finnish boys and girls aged 3–18 years and reported that PA behavior develops during childhood and tracks into adulthood with moderate-to-high stability (stability coefficients ≥ 0.60).

There is evidence from cross-sectional [10] and longitudinal studies [11] of an association between levels of PA and physical fitness. According to Caspersen et al. [12], physical fitness can be categorized into health- (e.g., cardiorespiratory endurance, muscle strength, etc.) and skill-related (e.g., speed, power, etc.) components of physical fitness. Wrotniak et al. [10] used accelerometers to objectively measure PA and observed that time in moderate-to-vigorous PA positively correlated with measures of muscular strength (standing broad jump; *r* = 0.40) and speed (running speed; *r* = − 0.36) in children aged 8–10 years. These results were confirmed in a 3-year longitudinal study in which positive associations were reported between time spent in moderate-to-vigorous PA measured through accelerometers and different components of physical fitness [i.e., muscle strength (handgrip, *β* = 0.06)], proxies of muscle power (vertical jump, *β* = 0.04) in children aged 6–12 years [11].

Evidence-based research indicates that physical fitness is a powerful marker of health in children and adolescents [6]. In particular, cardiorespiratory endurance [13] and muscle strength [8] have been found to be positively associated with markers of health in children and adolescents. In a systematic review, Mintjens et al. [13] reported that performance levels in cardiorespiratory endurance were positively related with body mass index, waist circumference, body fatness, and prevalence of metabolic syndrome. Furthermore, Garzia-Hermoso et al. [8] reported positive associations between measures of muscle strength and body mass index, skinfold thickness, insulin resistance, triglycerides, cardiovascular disease risk score, and bone mineral density. Testing of physical fitness is easy-to-administer, reliable and valid which is why it should be extensively implemented to receive information on performance development and health of children and adolescents [14].

There is already evidence available in the literature on secular declines in physical fitness of children and adolescents. Tomkinson et al. [15, 16] summarized the existing literature on this topic for the timespan between 1958 and 2003. They included physical fitness data for 25,000,000–50,000,000 children and adolescents aged 6–19 years living in 27 countries across five geographical regions. Over the entire analysis period (1958–2003), cardiorespiratory endurance declined by − 0.36% per annum (p.a.). A more in-depth analysis indicated that between 1958 and 1970, cardiorespiratory endurance improved by + 0.61% p.a. Thereafter, performance declined by − 0.54% p.a. Findings for proxies of muscle power and speed were different in as much as there was an overall positive trend for both qualities (proxies of muscle power: + 0.03% p.a.; speed + 0.04% p.a.) between 1958 and 2003. When looking at the period from 1958 to 1985, improvements were found for proxies of muscle power (+ 0.44% p.a.) and speed (+ 0.27% p.a.). These were followed by annual declines in proxies of muscle power (− 0.20% p.a.) and speed (− 0.08% p.a.). Recently, Tomkinson et al. [17] published an update on secular trends in cardiorespiratory endurance restricting the analysis to data on the 20-m shuttle run test that were published between 1981 and 2014. In their meta-analysis, Tomkinson et al. [17] summarized data for almost 1,000,000 children and adolescents aged 9–17 years living in 19 different countries. According to their results, the international rate of cardiorespiratory endurance decline has slowed and stabilized since the turn of the century [17]. Furthermore, a recently published systematic analysis of secular trends on handgrip strength including data for 2,000,000 children and adolescents aged 9–17 years living in 19 countries showed that the international rate of improvement in handgrip strength progressively increased between 1967 and 2017 [18].

In summary, there is evidence that physical fitness of children and adolescents (particularly cardiorespiratory endurance) has declined globally, particularly in western industrialized countries until the turn of the century and stabilized ever since. These trend analyses in physical fitness were the starting point to specifically promote PA and physical fitness in children and adolescents, especially by the WHO [19].

Given the effort of the WHO over the past years to promote PA and physical fitness in children and adolescents [19], it appears important to regularly update these secular trend analyses. Considering that those updates are available only for cardiorespiratory endurance using estimated $\dot{V}$O_2peak_ from the 20-m shuttle run test [17] and absolute muscle strength data from handgrip dynamometry [18], it is timely to re-analyze the literature and examine secular trends for other components of physical fitness such as relative muscle strength, proxies of muscle power, and speed. It is also important to broaden the perspective on cardiorespiratory endurance, i.e., to include also tests other than the 20-m shuttle run test in the analyses.

The aim of this systematic review was to provide an ‘update’ on secular trends in selected components of physical fitness (i.e., cardiorespiratory endurance, relative muscle strength, proxies of muscle power, speed) in children and adolescents aged 6–18 years. With reference to recent studies [17, 18] and due to ongoing public health efforts to promote PA and fitness [19], we hypothesized a positive trend in physical fitness development of children and adolescents over time. We additionally expected that the reported secular trends are heterogeneous both in direction and magnitude and specific to the respective physical fitness component under consideration. This could be due to a multitude of tests that were used to assess the different components of physical fitness.

## Methods

This systematic review was carried out in accordance with the Preferred Reporting Items for Systematic Reviews and Meta-Analysis (PRISMA) statement guidelines [20, 21].

### Literature Search

The authors conducted a systematic computerized literature search in the electronic databases PubMed and Web of Science to locate studies that explicitly reported secular trends in physical fitness of apparently healthy children and adolescents. The literature search period covered publications until April 2019. An updated search in July 2020 could not identify any additional hits. The following Boolean search strategy was used to identify studies related to secular trends in physical fitness of children and adolescents. Proximity operators (“*”) were applied to search for root words: ("physical fitness" OR “cardiorespiratory endurance” OR “muscular endurance” OR “muscular strength” OR “body composition” OR flexibility OR agility OR balance OR coordination OR speed OR power OR “reaction time”) AND (child OR children OR youth OR adolescent OR adolescents OR adolescence) AND ("secular change*" OR "secular trend*" OR "secular decline*" OR "temporal trend*" OR "temporal change*" OR "temporal decline*").

**Fig. 1:**
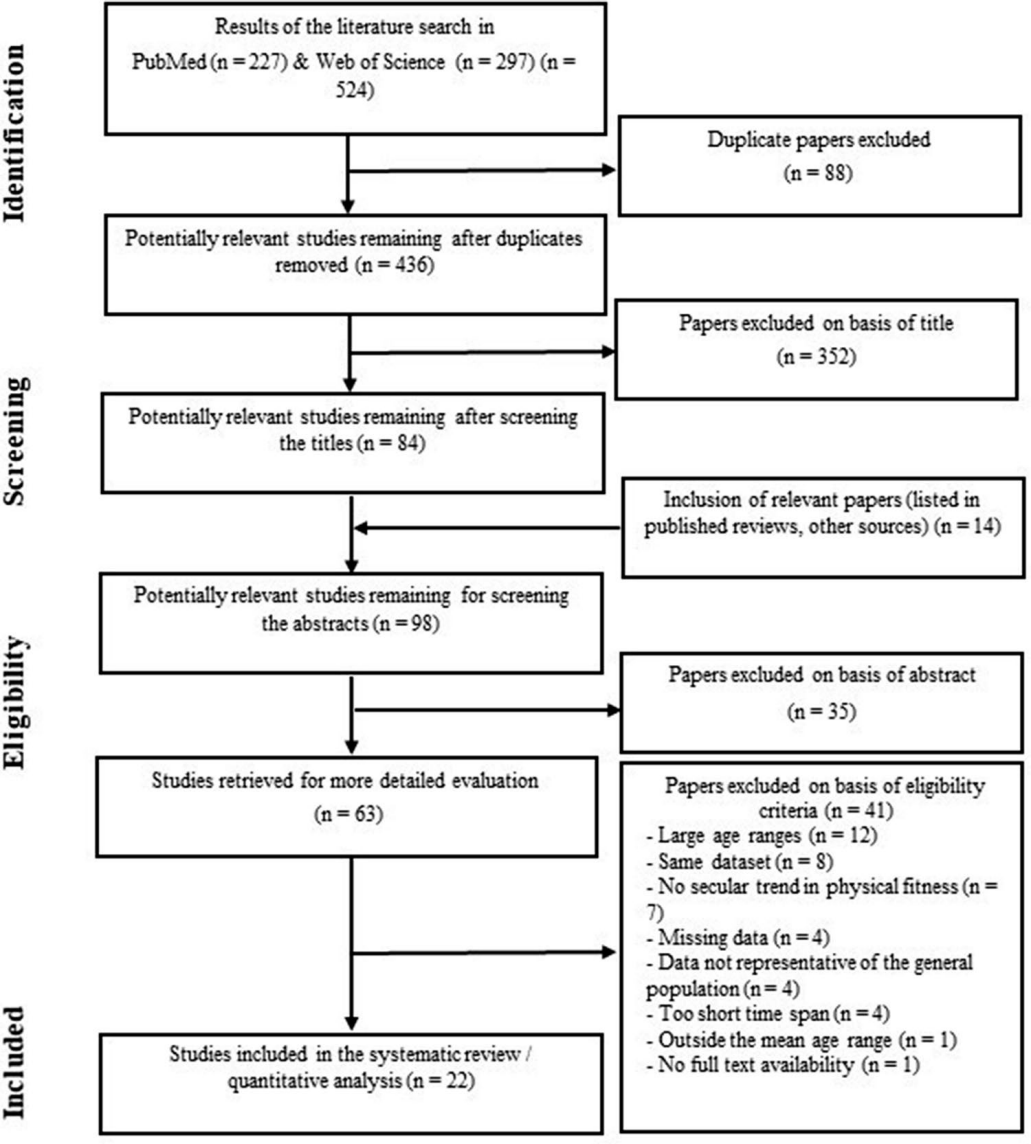
Flow chart illustrating the search and selection process of this systematic review

In addition, the following filters were activated for PubMed: species: humans; ages: birth–18 years (children), 13–18 years (adolescents). The applied search syntax for PubMed was adapted for the Web of Science database so that the abbreviation “TS = ” (for Topic) was placed in front of each bracket. Furthermore, reference lists of each article as well as relevant review articles and meta-analyses [15, 16, 22–31] were cross-referenced/screened to identify suitable adequate references to be included in this systematic review (see flow chart Fig. 1). We additionally scrutinized the database ‘Cochrane Library’, but could not identify any additional hits.

### Selection Criteria

Studies were integrated in this systematic review if they provided relevant information with regards to a modified PICOS approach [21]. The considered parameters were population, outcomes, and study design. The following predefined inclusion criteria were selected: (1) population: apparently healthy children and adolescents with a mean age of 6–18 years; differentiated age ranges not spanning more than 3 years (e.g., 12- to 14-year olds) according to Tomkinson et al. [32]; a sample distinguished by sex allowing sex-specific analysis as recommended by Tomkinson [16]; (2) intervention: not possible; (3) comparator: not possible; (4) outcomes: secular trends in selected physical fitness components (i.e., cardiorespiratory endurance, relative muscle strength, proxies of muscle power, speed) expressed as mean, standard deviation/standard error, and sample size according to these studies [15, 16, 23]; (5) study design: in accordance with Jürimäe et al. [33], studies which spanned at least two time points across a minimum of 5 years; only published articles/datasets with full-text availability; articles published in English or German. Studies were excluded for the following reasons: (1) population: patients or outside the mean age range of 6 to 18 years; large age ranges spanning more than 3 years (e.g., 12- to 15-year olds) [32]; children with an elite athletic background; children who live in specific areas (e.g., rural areas, Inuit) since their data are typically not representative of the general population [22]; a sample not distinguished by sex as recommended by Tomkinson [16]; (2) intervention: not possible; (3) comparator: not possible; (4) outcomes: assessment of secular trends not related to physical fitness; authors reported only aggregated physical fitness scores; missing data concerning means, standard deviation / standard error or sample size; (5) study design: samples which did not span at least two time points across a minimum of 5 years [33]; no full-text availability; articles published in languages other than English or German; data that were reported in several publications [32].

Two independent reviewers (TF, FA) screened potentially relevant papers by analyzing titles, abstracts, and full texts to determine study eligibility. If TF and FA did not reach agreement, UG was consulted for clarification.

### Coding of Studies

All included studies were coded for the following variables listed in Table 1. The author TF extracted the relevant data in an Excel spreadsheet from all included studies.

**Table 1:** Study coding

Nation	Australia, Brazil, Canada, Czech Republic, Denmark, England, Finland, Belgium (Flanders), Germany, Hungary, Lithuania, Netherlands, New Zealand, Norway, Portugal, Sweden, USA
Physical fitness components	Cardiorespiratory endurance, relative muscle strength, proxies of muscle power, speed
Chronological age	6–18 years
Sex	Boys and girls
Trends	Positive, negative, stagnation

If studies included several tests of the same physical fitness component, only one test was used according to the following ranking (Table 2). The list was created through expert rating based on the factors ‘practical relevance’ and ‘frequency of usage’ of the respective test.

**Table 2:** Classification of the ranked physical fitness tests used in the included studies

Cardiorespiratory endurance	Relative muscle strength	Proxies of muscle power	Speed
6-min run^e^	Leg lift test^e^	Standing broad jump^e^	20-m sprint^e^
9-min run^e^	Sit-up^a,e^	Sargent jump and reach test^e^	30-m sprint^e^
20-m shuttle run^e^	Push-up^b,e^		40-m sprint^e^
Cooper test (12 min)^e^	Bent arm hang^e^		45,7-m (50-yd) sprint^e^
1200-m run^e^	Pull-up^c,e^		50-m sprint^e^
1600-m run^e^	Arm-pull		4 × 9-m shuttle run
Maximal cycle ergometer^e^	Bench-press^d^		10 × 5-m shuttle run^e^
PWC 170 cycle ergometer^e^	Two-hand lift		
Submaximal cycle ergometer^e^			

^a^Maximum repetition; number at a rate of 25 lifts per minute or counts/30 s

^b^Counts/40 s

^c^Maximum repetition

^d^Number at a rate of 25 lifts per minute

^e^Tests were included in analyses

If relevant data (e.g., means) were only reported in figures or graphs, the program GetData-Graph-Digitizer (https://www.getdata-graph-digitizer.com/index.php) was used for data extraction purposes.

### Statistical Analyses

#### Computation of *z* scores for Components of Physical Fitness

Physical fitness was assessed through the fitness components cardiorespiratory endurance, relative muscle strength, proxies of muscle power, and speed. For measures of relative muscle strength, proxies of muscle power, and cardiorespiratory endurance, high scores indicate good and for measures of speed high scores indicate poor physical fitness. To facilitate readability, speed outcomes were multiplied by − 1. Thus, performance improvements over time were positive for all included outcome measures. The total sample size was calculated from the maximal sample sizes at age–time points–sex level for each included study.

As a common metric for tests, we converted means to *z* scores based on sample-weighted means and standard deviations for cells defined by study, test, and age of children. Specifically, means and standard deviations were pooled across sex and year within these cells. For three studies, standard deviations were computed from standard errors [34–36]; for two studies, the number of participants for subgroups was extrapolated from aggregate numbers [37, 38]. Of note, Spengler et al. [39] provided means and standard deviations for their study in response to a personal request. For pre- and post-processing of results, custom R code and (mainly) the following R packages were used: *tidyverse* [40], *remef* [41], *sjPlot* [42], *cowplot* [43], and *broom.mixed *[44]*.*

#### Statistical Inference with an Integrated Linear Mixed Model

We specified a linear mixed model (LMM) to estimate effects of sex and linear, quadratic, and cubic secular trends as nested under the four components of physical fitness (i.e., 8 × 4 = 32 fixed effects). For each observation, year of assessment was centred at 1990, i.e., at the midpoint of first-year assessments between 1974 and 2006 across studies. The nested model specification implies that for each of the four fitness components, we obtained an estimate for the year 1990 (i.e., equivalent to an intercept), an estimate for the main effect of sex, estimates for the secular trend (three parameters for linear, quadratic, and cubic component), and estimates for the interactions of sex and secular trend.

We tested the effects of sex and secular trends within each of the four physical fitness components because it was clear from the outset that the qualitative differences between the physical fitness components would yield trivial higher-order interactions. The limitation of the nested-model specification is that we did not obtain outcomes that report differences between these physical fitness components (e.g., sex × component or secular trend × component interactions). We considered separate LMMs for each physical fitness component. This approach, however, would not take dependencies between measures from different physical fitness components and between dependencies across years within studies into account. Moreover, the number of observations available for each LMM would substantially reduce statistical power for each of them.

The LMM was estimated with the *lmer()* function of the R-based *lme4* package [45, 46]. Model selection involved the determination of a random-effect structure that is supported by the data using random-effect principal component analysis [47, 48]. The final model included study-related variance components for cardiorespiratory endurance, relative muscle strength, sex, and linear yearly trends for cardiorespiratory endurance, relative muscle strength, and proxies of muscle power. The specification of correlation parameters for these variance components led to overparameterized models or did not significantly contribute to the goodness of fit as assessed with likelihood ratio tests. With this random-effect structure, none of the high-order interactions between sex and quadratic or cubic secular trends were significant.^1^ Therefore, we removed these eight fixed effects from the LMM. The protocol of model selection is available as Electronic Supplementary Material Appendix S2.

The LMM treated the 22 studies as levels of a random factor; they contributed a total of 652 observations. The LMM estimated 24 fixed effects, six variance components, and the residual variance (i.e., a total of 31 model parameters). A |*z*| value > 2.0 (i.e., alpha of 5%) was used as a criterion for rejection of the null hypothesis.

In response to reviewer requests, we specified two additional LMMs. First, we included age of children (dichotomized at 12.5 years) as an additional factor and documented this analysis in Electronic Supplementary Material Appendix S1. The second request was to address possible geographical/cultural differences between studies from different countries. This analysis is also reported in Electronic Supplementary Material Appendix S1.

## Results

### Study Characteristics

A total of 524 potentially relevant studies were identified in the electronic databases PubMed and Web of Science. Fourteen articles were found through other sources such as reference lists of relevant reviews articles or meta-analyses. After screening for titles, abstracts, and full texts, 22 studies were finally eligible for inclusion in this systematic review article. Figure 1 illustrates the respective flow chart.

Table 3 summarizes the characteristics of the included studies. Secular trends in physical fitness were analyzed for *N* = 96,522 children and adolescents aged 6–18 years living in 17 different countries mainly in high-income countries such as the US, Australia, and Europe. Sample sizes ranged between 41 and 2,153 participants. Sixteen of the 22 studies were carried out in Europe [34, 37–39, 49–60], two in Australia [35, 61], one in the US [36], one in Canada [62], one in New Zealand [63], and one in Brazil [64]. The time span varied from six to 35 years with a mean value of 20 years and a median of 21 years. Six studies reported data for several time points [34, 39, 49, 57, 58, 64].

**Table 3:** Summary of the studies used in this systematic review

References	Country	Time points Observational period in brackets (years)	Sex	Age range (years)	Range of sample sizes	Physical fitness component	Test(s)
Matton et al. [49]	Belgium (Flanders)	1972^a^/1980^b^, 2005 (33^a^/25^b^)	M, F	12–17^c^	161–2,153	Strength, speed	BAH, SHR10 × 5
Westerstahl et al. [50]	Sweden	1974, 1995 (21)	M, F	16	185–230	Strength, power, endurance	9 min, THL, SJAR, SU, BP
Reiff et al. [36]	USA	1975, 1985 (10)	M, F	10–17^c^	196–786	Strength, power, speed	PU, SHR4 × 9, 45,7 m, SBJ
Mészáros et al. [51]	Hungary	1975, 2000 (25)	M	10–13^c^	160–191	Speed, endurance	30 m, 1200 m
Krombholz [52]	Germany	1977, 2000 (23)	M, F	6–7^c^	80–220	Power	SBJ
Sedlak et al. [53]	Czech Republic	1977, 2012 (35)	M, F	6	133–178	Power	SBJ
Sziva et al. [38]	Hungary	1979, 2004 (25)	M	7–11^c^	152–158^d^	Endurance	12 min
De Moraes Ferrari et al. [64]	Brazil	1979, 1989, 1999, 2009 (30)	M, F	10–11	43–184	Endurance	SCE
Runhaar et al. [54]	Netherlands	1980, 2006 (26)	M, F	9–12^c^	41–505	Strength, power, speed	BAH, SHR10 × 5, LLT, AP, SJAR
Reed et al. [62]	Canada	1981, 2004 (23)	M, F	9–11^c^	252–2,151^d^	Endurance	SHR 20 m
Dollmann et al. [61]	Australia	1985, 1997 (12)	M, F	10–11^c^	277–499	Power, speed, endurance	1600 m, 50 m, SBJ
Hardy et al. [35]	Australia	1985, 2015 (30)	M, F	9–15^c^	109–546	Power	SBJ
Wedderkopp et al. [55]	Denmark	1986, 1998 (12)	M, F	9	279–670	Endurance	MCE
McAnally et al. [63]	New Zealand	1987, 2011 (24)	M, F	15	157–436	Endurance	SCE
Aaberge and Mamen [56]	Norway	1988, 2001 (13)	M, F	15	77–106	Power, endurance	SCE, SJAR
Pampakas et al. [37]	Hungary	1989, 2004 (15)	M	7–10,5^c^	136–147^d^	Endurance	12 min
Venckunas et al. [57]	Lithuania	1992, 2002, 2012 (20)	M, F	11–18^c^	58–598	Strength, power, speed, endurance	SBJ, SHR10 × 5, BAH, SU, SHR 20 m
Costa et al. [34]	Portugal	1996, 2001, 2006, 2011 (15)	M, F	10–11	229–262	Strength, power, speed	SU, SBJ, 40 m
Sandercock et al. [58]	England	1998, 2008, 2014 (16)	M, F	10–11	150–158	Endurance	SHR 20 m
Palomäki et al. [59]	Finland	2003, 2010 (7)	M, F	15–16	640–1,142	Endurance	SHR 20 m
Albrecht et al. [60]	Germany	2005, 2011 (6)	M, F	11–13	352–466	Strength, power, endurance	SBJ, PHU, PWC
Spengler et al. [39]	Germany	2006–2015 (9)	M, F	6–7	220–274	Strength, speed, endurance	6 min, PHU, 20 m

Studies were sorted chronologically according to the first year of measurement. The table illustrates the country of origin, the time points (years), the observational period (years), sex, the respective age range of the participating children and adolescents, the range of sample sizes, and the test(s) for which secular trends were reported

M = male, F = female, BAH = bent arm hang, THL = two hand lift, SU = sit-up, BP = bench-press, PU = pull-up, PHU = push-up, LLT = leg lift test, AP = arm-pull, SBJ = standing broad jump, SJAR = Sargent Jump and Reach, 20 m = 20-m sprint, 30 m = 30-m sprint, 40 m = 40-m sprint, 45,7 m = 45,7-m (50-yd) sprint, 50 m = 50-m sprint, SHR4 × 9 = 4 × 9-m shuttle run, SHR10 × 5 = 10 × 5-m shuttle run, 6 min = 6-min run, 9 min = 9-min run, 12 min = 12-min run (Cooper test), 1200 m = 1200-m run, 1600 m = 1600-m run, SHR 20 m = 20-m shuttle run, PWC = PWC 170 cycle ergometer, SCE = submaximal cycle ergometer, MCE = maximal cycle ergometer

^a^For male

^b^For female

^c^Data are available for each yearly age

^d^Authors only reported the whole sample size

To assess secular trends in physical fitness, 15 out of 22 studies used tests for cardiorespiratory endurance [37–39, 50, 51, 55–64], 11 used tests for proxies of muscle power [34–36, 50, 52–54, 56, 57, 60, 61], eight for relative muscle strength [34, 36, 39, 49, 50, 54, 57, 60], and eight for speed [34, 36, 39, 49, 51, 54, 57, 61]. A total of 26 different physical fitness tests were found in the included studies (Table 2).

Studies which assessed cardiorespiratory endurance included submaximal [56, 60, 63, 64] or maximal [55] tests on a cycle ergometer, the 20-m shuttle run test [57–59, 62] or time-related (6-min run test [39], 9-min run test [50], Cooper test [37, 38]) as well as distance-related cardiorespiratory endurance tests (1200-m run test [51], 1600-m run test [61]). Studies which assessed relative muscle strength comprised five different lower/upper limbs strength tests as well as tests for the assessment of trunk muscle strength. Accordingly, tests included the leg lift test [54], sit-up test [34, 50, 57], push-up test [39, 60], bent arm hang test [49], and pull-up test [36]. Studies which assessed proxies of lower limbs muscle power included either the standing broad jump test [34–36, 52, 53, 57, 60, 61] or the Sargent jump and reach test [50, 54, 56]. Studies which assessed speed comprised either linear sprint tests over short distances such as the 20-m sprint test [39], 30-m sprint test [51], the 40-m sprint test [34], the 45-m sprint test [36], the 50-m sprint test [61] or short shuttle runs such as the 10 × 5-m shuttle run test [49, 54, 57] (Table 3).

### Secular Trends and Effects of Sex for Components of Physical Fitness

The 24 fixed-effect LMM estimates relating to sex, secular trend, and their interactions for each of the four components are displayed in Table 4 along with standard error, *z* statistic, and *p* value. In general, we observed the expected higher scores for boys than girls in all panels with effects ranging between 0.094 (relative muscle strength; not significant with *p* < 0.07) and 0.44 (cardiorespiratory endurance, *p* < 0.001). Interpretation of polynomial trends, however, must start with the highest-order significant trend or its interaction with sex; lower-order trends and main effects are usually qualified by them. In the following, we report statistics for the critical terms (lifted from Table 4); statistics for all lower-order terms are reported in the table.

**Table 4:** Fixed-effects estimates of linear mixed model

Component	Fixed-effects estimates	Standard error	*z* values	*p* values
Cardiorespiratory endurance	0.32595	0.12234	2.66	**0.008**
Sex	0.44032	0.05348	8.23	** < 0.001**
Year (linear)	− 0.02419	0.01027	− 2.36	**0.018**
Year (quadratic)	− 0.00365	0.00084	− 4.35	** < 0.001**
Year (cubic)	0.00012	0.00003	3.94	** < 0.001**
Sex × year (linear)	− 0.00796	0.00243	− 3.28	**0.001**
Relative muscle strength	− 0.17252	0.07174	− 2.40	**0.016**
Sex	0.09356	0.05087	1.84	0.066
Year (linear)	0.01095	0.00720	1.52	0.128
Year (quadratic)	0.00067	0.00033	2.069	**0.039**
Year (cubic)	− 0.00004	0.00002	− 2.14	**0.032**
Sex × year (linear)	0.00066	0.00184	0.36	0.721
Proxies of muscle power	0.09430	0.03376	2.79	**0.005**
Sex	0.36078	0.04894	7.37	** < 0.001**
Year (linear)	− 0.00093	0.00536	− 0.17	0.863
Year (quadratic)	− 0.00060	0.00030	− 2.04	**0.042**
Year (cubic)	0.00001	0.00002	0.70	0.486
Sex × year (linear)	0.00474	0.00169	2.80	**0.005**
Speed	0.03498	0.03732	0.94	0.349
Sex	0.18428	0.05015	3.67	** < 0.001**
Year (linear)	− 0.01822	0.00372	− 4.90	** < 0.001**
Year (quadratic)	− 0.00044	0.00021	− 2.11	**0.035**
Year (cubic)	0.00006	0.00001	4.60	** < 0.001**
Sex × year (linear)	0.00277	0.00180	1.54	0.125

**Fig. 2:**
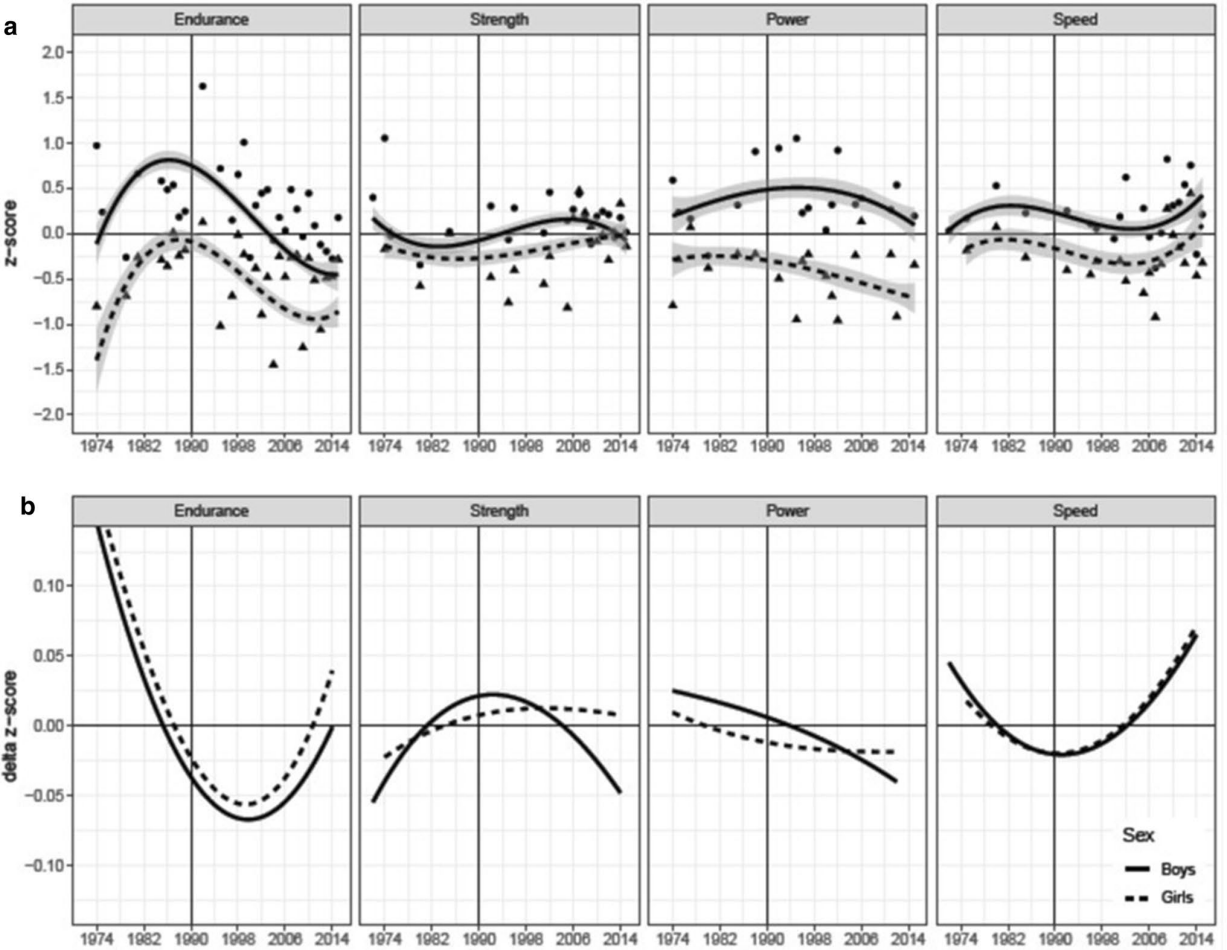
**a** Secular trends for cardiorespiratory endurance, relative muscle strength, proxies of muscle power, and speed for standardized scores. Positive scores indicate better physical fitness in units of the standard normal distribution (*z* scores) relative to a grand mean of zero estimated for the year 1990. Dots represent the observed study means; the line is a third-order polynomial regression based on partial effects predicted from data and linear mixed model parameters (see Table 4); shaded areas represent 95% confidence intervals for the regression lines. **b** Numeric approximations of first derivatives (delta *z* score) of corresponding functions in panel a (i.e., local changes in the direction of the function). Minima and maxima in panel a correspond to zero-crossings in **b**; inflection points in cubic functions in panel a correspond to minima and maxima of first derivatives in **b**

The coefficients define the functions that generate the secular trends shown in Fig. 2a. Significant effects are in bold

Data and model parameters (up to the highest-order significant term) were used to generate the partial-effect functions for secular trends shown in Fig. 2a. Partial effects no longer contain effects due to differences between studies in cardiorespiratory endurance, relative muscle strength, sex, or due to linear yearly trends for cardiorespiratory endurance, relative muscle strength, and proxies of muscle power. Points in the figure represent the observed sex × year means that result from averaging *z* scores across observations within age × study/test cells. Note that corresponding means predicted by data and model parameters (i.e., the values underlying the function fit) would be closer to the function due to LMM shrinkage (i.e., adjustments for variability, number of observations, and deviation from the Grand Mean). Nevertheless, observed means are also in good correspondence with partial-effect *Z* functions.

Minima, maxima, and—for cubic *Z* functions—inflection points provide critical information about secular trends. The years at which they occur are directly visible in the first derivative delta *z* score of such function. They were computed with a numeric approximation depicting the instantaneous rate of change (see Fig. 2b). Specifically, minima and maxima of the *z* score function in a-panels are at the years at which the delta *z* score function in *b* panels crosses the horizontal zero-line; inflection points of cubic *z* score functions are at years with minima and maxima of delta *z* score functions.^2^ With these advanced organizers, we turn to the component-specific secular trends and how they differ for boys and girls.

#### Cardiorespiratory Endurance

The LMM included 168 observations for cardiorespiratory endurance. The critical source of variance is the positive cubic trend (*b* = 0.00012, *z* = 3.94, *p* < 0.001; see Fig. 2a, cardiorespiratory endurance panel). Children’s cardiorespiratory endurance increased until 1986 and then decreased until around 2010–2012 with a negative inflection point in 2000. Since 2010, cardiorespiratory endurance may have stabilized or even tended to improve again (see zero-crossings and minimum in cardiorespiratory endurance panel Fig. 2b for identification of years). The interaction between sex and the linear trend for years is the source for a tendency towards smaller differences between boys’ and girls’ cardiorespiratory fitness or a steeper decline for boys than girls (*b* = − 0.00796, *z* = − 3.28, *p* = 0.001).

In terms of effect magnitude (i.e., the difference between maximum and minimum *z* scores across years), cardiorespiratory endurance exhibited very large secular trends with a range of about 1.5 standard deviations for both boys and girls. These effects are by far the largest of the four fitness components.

#### Relative Muscle Strength

The LMM included 156 observations for relative muscle strength. The critical source of variance is the cubic secular trend for all children (see Fig. 2a, relative muscle strength panel); *b* = − 0.00004, *z* = − 2.14, *p* = 0.032. There are no significant effects associated with sex, but the trend is clearer for boys. An initial decline with a local minimum in 1982 is followed by an increase to a local maximum in 2006 with a positive inflection point in 1990. The magnitude of the secular trend is small; scores move consistently within a narrow 0.50 *z* difference band.

#### Proxies of Muscle Power

The LMM included 164 observations for proxies of muscle power. The interaction between sex and the linear trend across years translates into shifting a very shallow negative quadratic curve (*b* = − 0.0006, *z* = − 2.04, *p* = 0.042) to different peaks in 1982 for girls and 1994 for boys; *b* = 0.00474, *z* = 2.80, *p* = 0.005. With 0.5, the effects are in the small-to-medium range.

#### Speed

The LMM included 164 observations for speed. Boys and girls exhibit a similar shape of their secular speed function (see also the close to identical delta *z* score functions in the speed panel of Fig. 2b). The shape of the function is defined by a significant negative linear (*b* = − 0.01822, *z* = − 4.90, *p* < 0.001) and a significant positive cubic (*b* = 0.00006, *z* = 4.60, *p* < 0.001) trend. The first local peak was in 1980 (a very shallow one); the negative inflection point was in 1990; speed bottomed out in 2002 and has been rising since then. Effect sizes are about 0.50 for both groups.

#### Effect Sizes and Conditional Means

The four components of physical fitness varied qualitatively with respect to the shape of their associated secular trends and differed between boys and girls in cardiorespiratory endurance and proxies of muscle power. They also differed in effect sizes. Effect sizes were large for cardiorespiratory endurance (1.5 units of standard deviation) and small-to-medium (0.5 units of standard deviations) for the other three components. Importantly, the partial-effect functions are corrected for various sources of heterogeneity between studies as described in the following.

LMMs afford tests of heterogeneity of studies with estimates of variance components (VCs) as model parameters that account for differences between studies beyond the residual error variance. Specifically in the present data, there was no evidence for speed-related and proxies-of-muscle-power-related VCs, but there were significant VCs for cardiorespiratory endurance (0.36),^3^ relative muscle strength (0.13), sex (0.18), and three linear yearly trends associated with cardiorespiratory endurance (0.03), relative muscle strength (0.013), and proxies of muscle power (0.011); the residual standard deviation was 0.25. Thus, there was considerable heterogeneity between studies and it was largest for cardiorespiratory endurance.

**Fig. 3:**
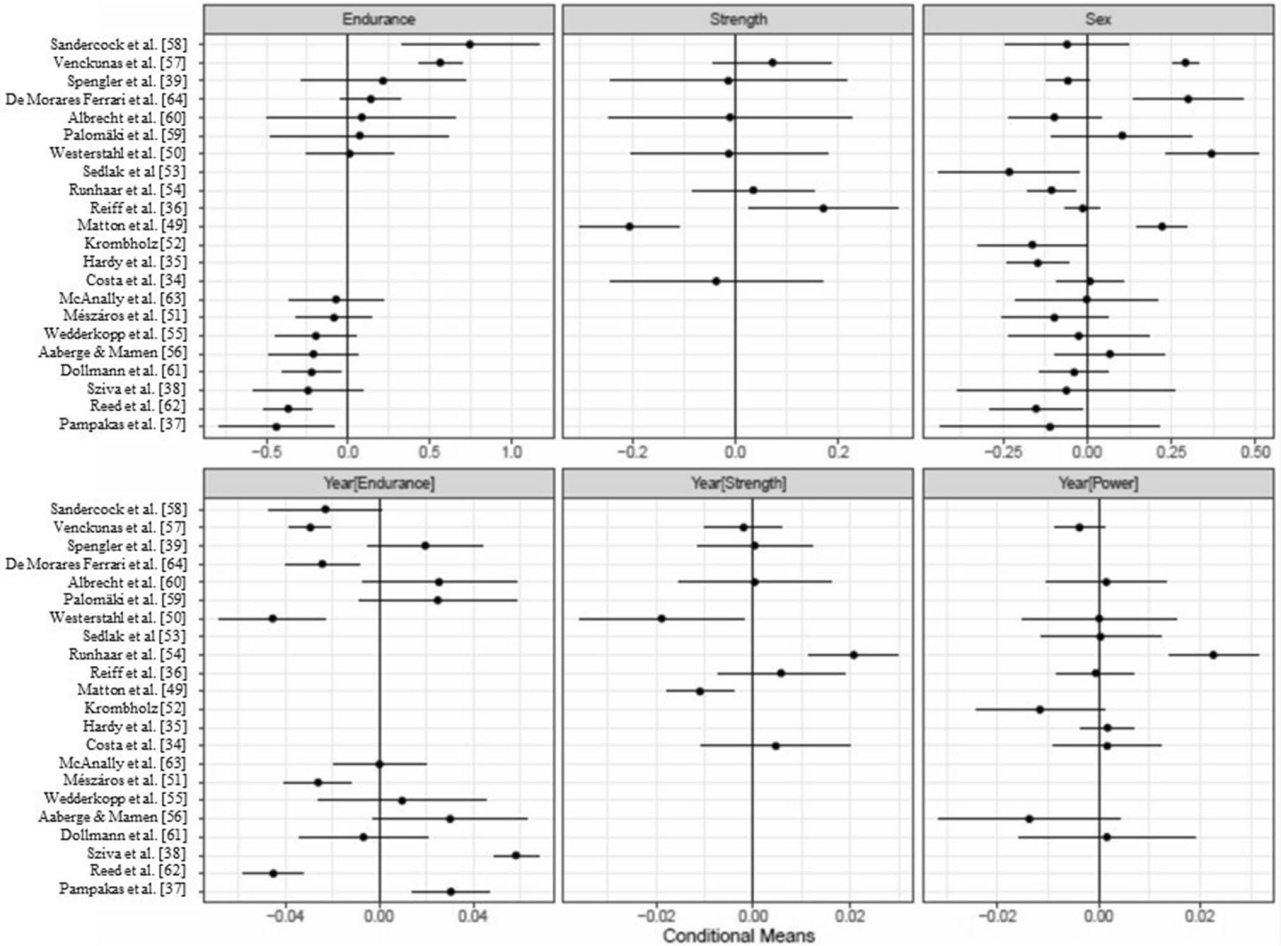
Conditional means of six significant variance components in the linear mixed model

Data and model parameters can be used to generate predictions of conditional means at the level of studies for components of physical fitness with reliable inter-study differences. These predictions are shown in Fig. 3 along with intervals of ± 2 conditional standard deviations (~ 95% confidence intervals), based on the conditional variance–covariance matrices of the random effects returned by the *ranef(model, condVar* = *TRUE)* command of the *lme4* R package [45].

The conditional means differ from the observed means because they are shrunken towards the estimated population mean, conditional on (a) the distance of the observed from the grand mean, (b) the variance of the observed mean, and (c) the number of observations. Studies with confidence intervals completely to the left or the right of the vertical zero line (representing the grand mean), contributed significantly to heterogeneity. The major determinants of the width of the confidence intervals are the number of observations and their variance. For example, Veckunas et al. [57] contributed 48 and Sandercock et al. [58] four observations to the cardiorespiratory endurance component, accounting for the large difference between their respective intervals. Studies without an entry in a panel did not contribute observations to these components or their conditional mean was shrunk to the population mean. Taking into account this between-study heterogeneity was critical for the shape of the partial-effects functions in Fig. 2a.

#### Observation-Level Model Residuals

**Fig. 4:**
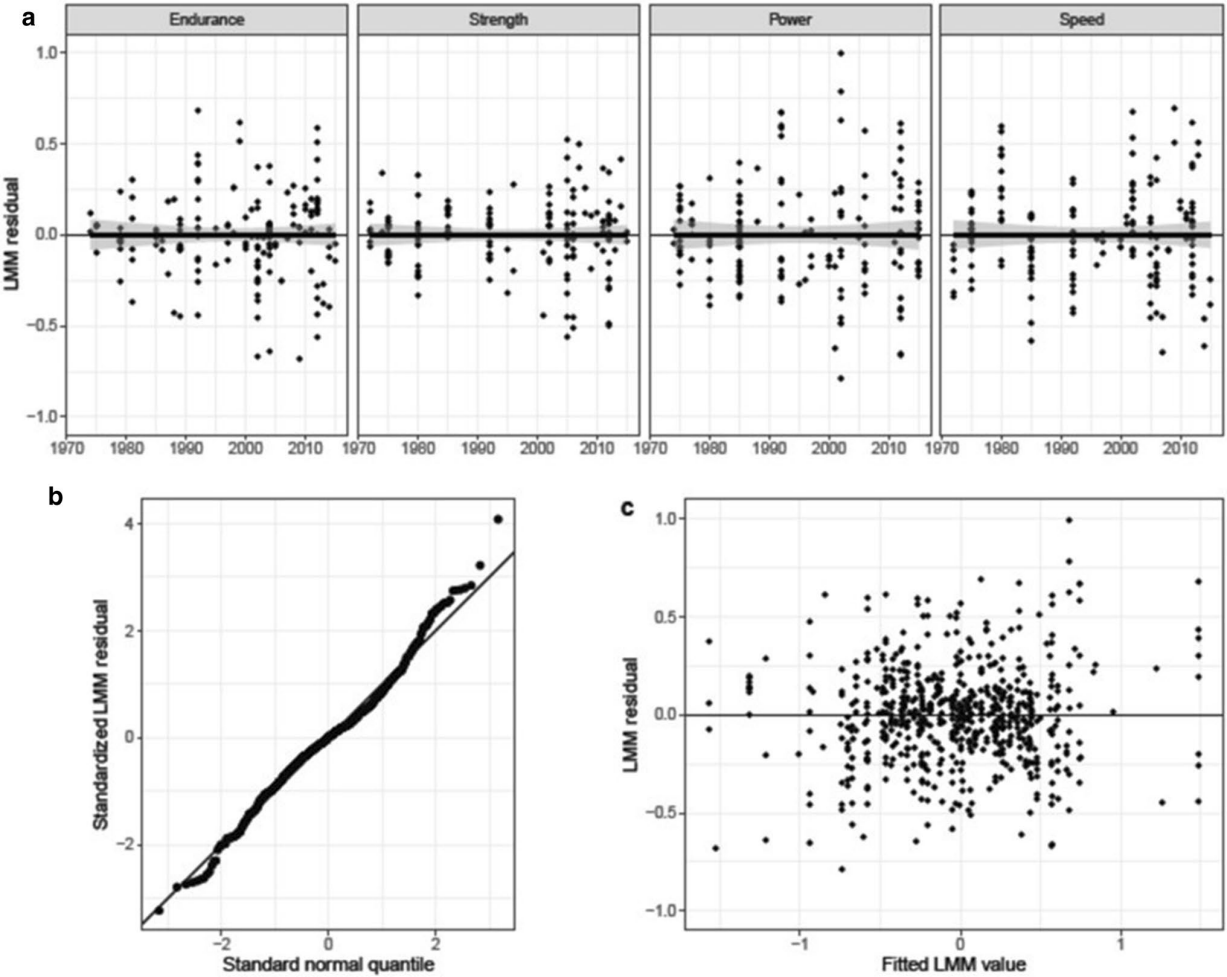
Observation-level residuals of linear mixed model (LMM). **a** LMM residuals over year by physical fitness component; **b** standardized LMM residuals over theoretical quantiles of the standard normal distribution; **c** LMM residuals over fitted values

The quality of a model fit depends on distributional characteristics of the observation-level model residuals, most importantly that they are normally distributed and homoscedastic across the range of fitted values. Figure 4 displays a few diagnostic statistics (a: LMM residuals over year by component; b: standardized LMM residuals over theoretical quantiles of the standard normal distribution; c: LMM residuals over fitted values).

The plots provide no evidence for violations of model assumptions. Somewhat surprisingly, there was no need for dealing with outliers. We interpret this as support for the quality of data selection, effectiveness of controlling for study heterogeneity as well as the selection of tests and their assignment to the four components of physical fitness.

## Discussion

The aim of this systematic review was to provide an update on secular trends in selected components of physical fitness of children and adolescents aged 6–18 years. The main findings of this systematic review including 22 studies and 652 observations were (a) a large initial increase and an equally large subsequent decrease between 1986 and 2010–12 in cardiorespiratory endurance; the decrease appears to have reached a floor for all children between 2010 and 2015, but it was steeper for boys than girls, (b) a general trend towards a small increase in relative muscle strength, (c) an overall small negative quadratic trend for proxies of muscle power; and (d) a small-to-medium increase in speed since 2002.

### Secular Trends and Effects of Sex for Components of Physical Fitness

In this systematic review, we provide new information on secular trends in physical fitness of children and adolescents (i.e., cardiorespiratory endurance, relative muscle strength, proxies of muscle power, and speed). There is evidence that cardiorespiratory endurance particularly declined between 1986 and 2010–12. Of note, it seems that the decline diminished during recent years for boys and girls. This finding confirms a recently published update by Tomkinson et al. [17] for cardiorespiratory endurance which showed that the international rate in cardiorespiratory endurance decline has slowed, but stabilized since the turn of the century for both boys and girls. Furthermore, Tomkinson et al. [17] reported that declines in cardiorespiratory endurance were larger for boys compared to girls which is also in line with our results.

These corresponding findings between our systematic review and the analysis of Tomkinson et al. [17] emphasize that the two reviews complement each other despite their different methodological approaches. While Tomkinson et al. [17] focused on the 20-m shuttle run, we additionally included data for time- and distance-related cardiorespiratory endurance tests. Tomkinson et al. [17] used the performance data from the 20-m shuttle run test and the equation provided by Léger and colleagues [65] to estimate $\dot{V}$O_2peak_ from 20-m shuttle run performance for children and adolescents aged 8–19 years, irrespective of sex. In contrast, we used *z* scores as a common metric for all included tests.

Tomkinson and Olds [15] outlined that secular trends in physical fitness are affected by several aspects such as changed social, behavioral, physical, psychosocial, and physiological factors. In particular, psychosocial factors such as motivation, one’s willingness to push to maximally, and pacing strategies may have played a role, but unfortunately cannot be controlled. There are several reasons why especially fat mass and BMI may influence cardiorespiratory fitness. First, two studies reported that 40% [66]–70% [67] of the changes in cardiorespiratory endurance can be explained by changes in BMI. Second, performance in weight-bearing activities such as running over longer distances, etc. is negatively influenced by a high fat mass [28]. Cureton et al. [68] showed that gains in weight/higher fat mass significantly increased the energy cost during running. Therefore, the $\dot{V}$O_2max_ (expressed relative to body mass) has a lower asymptote. Third, trends in cardiorespiratory endurance (decline which was recently mitigated) coincide with international trends of overweight and obesity. The NCD Risk Factor Collaboration [69] reviewed an increasing prevalence in overweight and obesity in school-aged children worldwide between 1975 and 2016, but stated that the trend has recently flattened, albeit at a high level, especially in high-income countries.

Another factor that might affect cardiorespiratory endurance is the level of PA. Although evidence-based information about secular trends in PA is missing [70], a recently published study by Guthold et al. [4] stated that in 2016 about 80% of children and adolescents (girls: 84.7% and boys: 77.6%) worldwide did not meet the recommended 60 min at moderate-to-vigorous PA per day proposed by the WHO. However, compared with data for 2001, PA levels have been relatively stable in girls over the past 15 years (85.1%), and have slightly improved in boys (80.1%). Keeping in mind the causal interaction between PA and physical fitness [11], the low(er) levels of PA might lead to a diminished exercise stimulus and consequently to reduced cardiorespiratory endurance [66].

Our update provided information on secular trends in relative muscle strength indicating a general trend towards a small increase in fitness related to relative muscle strength. In this respect, our systematic review also complements a recently published analysis conducted by Dooley et al. [18] about secular trends in absolute muscle strength using handgrip data. They showed that the international rate of improvement in handgrip strength progressively increased between 1967 and 2017 in 2,000,000 children and adolescents aged 9–17 years living in 19 countries.

With respect to proxies of muscle power, our systematic review revealed a small negative quadratic trend and with respect to speed a small-to-medium increase in recent years. Here, we describe some differences from an earlier comprehensive review that analyzed similar measures of speed and proxies of muscle power. Tomkinson [16] summarized the existing literature on physical fitness of children and adolescents between 1958 and 2003. He included proxies of muscle power and speed data for 25,000,000 children and adolescents aged 6–19 years living in 27 countries across five geographical regions between 1958 and 2003. For proxies of muscle power, Tomkinson [16] reported a decline starting in 1985 and no evidence for differences between boys and girls. We observed a decline starting in 1995 after a previous increase for boys and a flattening for girls. According to the Tomkinson review [16], speed has flattened since about 1985 after a previous slight increase for boys and girls. In contrast, we observed an initial increase which changed to a decline at about 1982 until 2002 for boys and girls. We note that our effects are in the small-to-medium range and that we had fewer studies contributing to these two fitness components than for cardiorespiratory endurance. Tomkinson [16] considered a much larger data base including the grey literature, many more children and adolescents and many more countries. The differences may well be due to differences in methods and study design.

Compared to secular trends in cardiorespiratory endurance (1.5 SD for boys and for girls), smaller trends were observed for relative muscle strength, proxies of muscle power, and speed (0.5–1.0 SD). The reason for the observed different effects might be the varying influence of fat mass/BMI on components of physical fitness. Tomkinson et al. [28] examined to what extent fat mass/BMI influences cardiorespiratory endurance, proxies of muscle power, and speed. Fat mass/BMI correlated stronger with cardiorespiratory endurance (1600 m run) than with a proxy of muscle power (standing broad jump) or speed (50-m sprint). The correlations were *r* = − 0.52 for cardiorespiratory endurance, *r* = − 0.20 for proxies of muscle power, and *r* = − 0.24 for speed [28]. Furthermore, Tomkinson et al. [28] stated that for fat-free mass, the effect is the opposite. The correlations between fat-free mass and proxies of muscle power (*r* = 0.59) and speed (*r* = 0.57) were stronger than for cardiorespiratory endurance (*r* = 0.21). Based on our findings and the available data, we cannot conclude whether it is a matter of fitness or fatness. When taking the work of Tomkinson et al. [28] into account, it seems that fatness plays a role particularly in cardiorespiratory endurance.

### Overall Interpretation and Implications

Overall, this systematic review demonstrated that there is no generic secular trend across the different physical fitness components and years because of significant cubic secular trends in measures of cardiorespiratory endurance, relative muscle strength, proxies of muscle power, and speed. Therefore, physical fitness components must best be analyzed separately because the components showed different patterns of secular trends over time. We recommend to regular examination of secular trends according to the specific physical fitness components and the monitored time span to detect changes in physical fitness as early as possible. Schools offer a suitable setting for the assessment of physical fitness because they reach all children during their development, growth, and formation of habits.

Because of the different trends in physical fitness observed in this systematic review and the literature, we recommend further initiatives in PA and fitness promotion for children and adolescents. More specifically, public health efforts should focus on exercising cardiorespiratory endurance and muscle strength because cardiorespiratory endurance [13] and muscle strength [8] have been reported to be positively associated with markers of health (i.e., body mass index, waist circumference, body fatness, bone mass) in children and adolescents. In addition, sufficient levels of muscle strength are a prerequisite for motor skill learning [71].

In this context, schools have a responsibility for promoting a physically active lifestyle because this is the only setting that reaches all children regardless of their socioeconomic background [72]. Therefore, structural curricula such as active recess, active education or active transportation to school, etc. are reasonable factors to reduce sedentary behavior and promote PA [73, 74]. It is crucial that parents and families are also included in these efforts [59]. Furthermore, the role of physical education classes needs to be strengthened to enable enhanced PA and fitness levels [73–75]. Several studies verify the positive impact of resistance training [76–80] and endurance training [81, 82] on children’s physical fitness. Based on this research, muscle strengthening and aerobic exercises can be included in the curricula as several studies [83–85] indicated that well-structured and well-designed physical education lessons can have a positive impact on the physical fitness of children and adolescents. In addition, Faigenbaum and Bruno [86] stated that children and adolescents who are regularly engaged in PA (e.g., through physical education, etc.) were more eager to develop complex movements and sport skills (e.g., in a sport club or free play).

### Limitations

This systematic review has some limitations. First, biological maturity or anthropometric data (e.g., body mass or body height) were not considered in the analysis because this information was not available in most of the included studies. There is strong evidence that physical fitness is related to biological maturity as more mature children and adolescents outperform less mature children and adolescents in physical fitness [87]. Furthermore, studies [88, 89] indicated a trend towards earlier maturation (age of puberty onset) over the last few centuries which is accompanied by an increase in physical fitness which may have influenced and altered secular trends. Future studies of secular trends in physical fitness of children and adolescents should always report the biological maturity status if possible. Second, every included study used different study designs (tests, instruments, methods, environmental conditions, etc.) for measuring physical fitness which resulted in inter-study variance especially for cardiorespiratory endurance and relative muscle strength. To better evaluate secular trends in physical fitness, future studies are advised to use identical test procedures among representative samples of children and adolescents worldwide [90]. Third, secular trends were mainly reported for high-income countries such as the United States, Australia, and Europe. Future studies should also be conducted in middle- and low-income countries using simple, cheap, valid, and reliable physical fitness measures. Fourth, the included studies are usually not representative of the population of their countries. Future studies should collect country-specific representative data. Fifth, all included studies reported mean values of secular trends. However, trends in mean values might be systematically biased if there have been concurrent trends in skewness whereas median values will not be biased. Future studies should also relate to measures of centrality (e.g., medians), variability (e.g., standard deviations), and asymmetry (e.g., skewness). Sixth, we do not claim that our systematic review was exhaustive; grey literature, project reports or unpublished work were not considered. Finally, an assessment of risk of bias was not conducted. Accordingly, future studies should include risk of bias assessment in their analysis.

## Conclusions

This systematic review documents a large initial increase and an equally large subsequent decrease for cardiorespiratory endurance starting in 1986 and lasting until 2010–12. The decrease appears to have reached a floor for all children between 2010 and 2015. Measures of relative muscle strength showed a general trend towards a small increase. Measures of proxies of muscle power indicated an overall small negative quadratic trend. For measures of speed, a small-to-medium increase was observed since 2002. Because of the different trends in physical fitness, we recommend further initiatives in PA and fitness promotion for children and adolescents. More specifically, public health efforts should focus on exercising cardiorespiratory endurance to prevent adverse health effects (i.e., overweight, obesity) and muscle strength to lay a foundation for motor skill learning. Furthermore, studies are needed that examine dose–response relations for muscle and bone strengthening exercises in children and adolescents.

## Electronic supplementary material

Below is the link to the electronic supplementary material.

Supplementary file1 (DOCX 215 kb)

Supplementary file2 (XLSX 51 kb)

## Footnotes

1 Fixed effects were not considered and not even inspected during selection of the random-effects structure supported by the data.

2 These relations derive from the power rule of differentiation; of relevance here are: f(x) = x—> f’(x) = constant, f(x) = x^2^—> f’(x) = 2x, and f(x) = x^3^—> f’(x) = 3x^2^.

3 Values are variances expressed as standard deviations (i.e., the square-root of the estimated VC).
