# Supplementary material for: An Update on Secular Trends in Physical Fitness of Children and Adolescents from 1972 to 2015: A Systematic Review
Source: Sports Med. 2020 Nov 7;51(2):303–20. doi: 10.1007/s40279-020-01373-x (PMC7846517; doi:10.1007/s40279-020-01373-x)
Supplement: Supplementary file 1 — Supplementary file1 (DOCX 215 kb) [file 40279_2020_1373_MOESM1_ESM.docx]

**An update on secular trends in physical fitness of children and adolescents from 1972 to 2015: A systematic review**

**Electronic Supplementary Material Appendix S1 & S2 – Sports Medicine**

Thea Fühner^1^

Reinhold Kliegl^1^

Fabian Arntz^1^

Susi Kriemler^2^

Urs Granacher^1^

^1^Division of Training and Movement Sciences, Research Focus Cognition Sciences, University of Potsdam, Am Neuen Palais 10, Building 12, 14469 Potsdam, Germany

^2^Epidemiology, Biostatistics and Prevention Institute, University of Zurich, Hirschengraben 84, 8001 Zurich, Switzerland

**Corresponding author and contact details**

Prof. Urs Granacher, PhD

Division of Training and Movement Sciences

Research Focus Cognition Sciences

University of Potsdam

Am Neuen Palais 10, Building 12

14469 Potsdam

Germany

Tel: +49-331-977 1543

E-mail address: urs.granacher@uni-potsdam.de

ORCID ID: orcid.org/0000-0002-7095-813X

**Electronic Supplementary Material Appendix S1**

In the electronic supplementary material, we document two additional analyses carried out in response to reviewer requests the effects of children’s age and the effect of geographic/cultural region. We report two linear mixed models (LMM) that are extensions of our reference LMM. The LMM with age includes additional fixed effects; the LMM with region includes an additional random factor.

**Children’s age**

Are the secular effects different for young and old children? We had not planned to investigate this research question in the systematic review. Therefore, we offer the following analysis as an exploratory one that is worth being followed up in future research. We had standardized within age groups. Therefore, we did not expect a main effect of age, but there may still be interactions with sex or the secular trend. We proceeded in three steps. First, we transformed age to a two-level factor (young: ≤ 12 years; old: ≥ 13 years) and faceted Figure 2a in our systematic review on this variable. The results for young children are shown in the top row and the results for old children in the bottom row of Electronic Supplementary Material Figure S1.


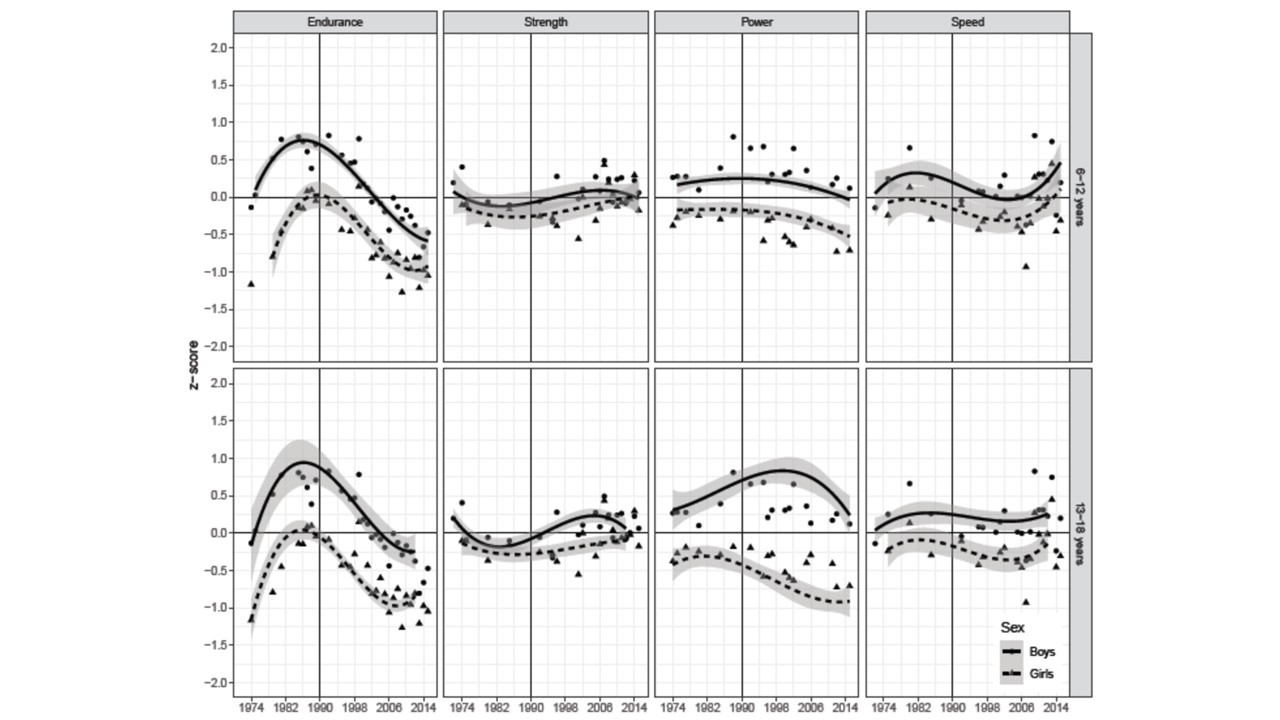


**Fig. S1**  Faceting of panels of Figure 2 in our systematic review by age of children. Young (≤ 12 years) and old children (≥ 13 years) exhibit the same qualitative secular profiles. Fits are based on the cubic linear mixed model *without* age as a covariate (based on model estimates reported in Table 4 in our systematic review)

We submit that the secular trends based on the reference LMM are *qualitatively* *similar* for young and old children; actually, the similarity lives up to what we would expect from a cross-validation. Tomkinson et al. [1], coding children’s age also as dichotomous factor, reported similar secular trends for young and old children for the 20-m shuttle run test. However, there is also at least one visible age x sex x secular trend interaction (e.g., the overall negative quadratic trend for proxies of muscle power is maintained for young children, but this secular trend appears to follow a negative cubic function for older girls with an inflection at around 1998 and minimum around 2010).

Can we recover this and possibly other interactions in an extended LMM? In the second step, we included dichotomous age as an additional main effect in the reference model, that is we kept the random-effect structure with six variance components and estimated the age effect for each of the four components. As expected, there was no significant increase of goodness-of-fit over the default LMM; LRT-based χ^2^ (4) = 7.3, p = 0.12. In a third step, we added all age x sex, age x secular trend, and age x sex x secular trend interactions, estimating 24 additional fixed effects (i.e., a total of 48) in comparison with the reference LMM. Somewhat surprisingly, this new LMM was supported by the data (i.e., not overparameterized or degenerate). Moreover, the goodness of fit was significantly better than the one for the reference LMM; LRT-based χ^2^ (24) = 205.48, p < 0.001.

Electronic Supplementary Material Table S1 displays the estimates and z-statistics in two super-columns along with the z-statistics of the reference LMM copied from Table 4 in our systematic review in the leftmost column. The next two columns report these statistics for main effects/interactions and z-values for the LMM with age and the final two columns the statistics for the corresponding age x fixed-effect interaction terms and z-values.

**Table S1** Fixed-effects estimates of LMM with age (≤ 12 years; ≥ 13 years)

| **Predictor** | **Reference LMM** | **LMM with Age** | | | |
| --- | --- | --- | --- | --- | --- |
|  | **z-value** | **Fixed effect estimate** | **FE**  **z-value** | **Age x FE estimate** | **Age x FE**  **z-value** |
| **Cardiorespiratory endurance** | **2.66** | 0.33670 | **2.93** | -0.12101 | **-2.04** |
| Sex | **8.23** | 0.50893 | **11.93** | -0.10702 | **-3.25** |
| Year (linear) | **-2.36** | -0.02632 | **-2.42** | 0.01449 | **2.41** |
| Year (quadratic) | **-4.35** | -0.00389 | **-4.85** | 0.00053 | 1.30 |
| Year (cubic) | **3.94** | 0.00014 | **4.84** | -0.00006 | **-3.04** |
| Sex x year (linear) | **-3.28** | -0.00803 | **-3.86** | -0.00217 | -1.05 |
| **Relative muscle strength** | **-2.40** | -0.19307 | **-2.46** | 0.00435 | 0.12 |
| Sex | 1.84 | 0.14846 | **3.67** | -0.07196 | **-3.42** |
| Year (linear) | 1.52 | 0.01371 | 1.82 | -0.00521 | -1.32 |
| Year (quadratic) | **2.07** | 0.00083 | **2.48** | -0.00013 | -0.63 |
| Year (cubic) | **-2.14** | -0.00005 | **-2.93** | 0.00002 | 1.34 |
| Sex x year (linear) | 0.36 | 0.00099 | 0.62 | -0.00323 | **-2.31** |
| **Proxies of muscle power** | **2.79** | 0.09212 | **3.19** | -0.05027 | -1.74 |
| Sex | **7.37** | 0.41126 | **10.72** | -0.23079 | **-11.70** |
| Year (linear) | -0.17 | -0.00150 | -0.28 | -0.00298 | -0.77 |
| Year (quadratic) | **-2.04** | -0.00053 | -1.93 | 0.00015 | 0.73 |
| Year (cubic) | 0.70 | 0.00001 | 0.74 | 0.00000 | 0.31 |
| Sex x year (linear) | **2.81** | 0.00567 | **3.96** | -0.00402 | **-3.09** |
| **Speed** | 0.94 | 0.02286 | 0.72 | -0.00296 | -0.10 |
| Sex | **3.67** | 0.23591 | **5.98** | -0.08104 | **-3.98** |
| Year (linear) | **-4.90** | -0.01676 | **-5.25** | -0.00435 | -1.37 |
| Year (quadratic) | **-2.11** | -0.00033 | -1.81 | 0.00004 | 0.22 |
| Year (cubic) | **4.60** | 0.00005 | **4.61** | 0.00001 | 0.75 |
| Sex x year (linear) | 1.54 | 0.00328 | **2.14** | -0.00258 | -1.87 |

Note. Reference linear mixed model (LMM) (left column): z-values copied from Table 4 in our systematic review. LMM with age: Fixed effects and z-values for terms w/o age (columns 2 and 3), age x fixed-effect interaction estimates (columns 4 and 5). Significant effects are set in bold.

As this is an exploratory analysis, we do not attempt an exhaustive interpretation of similarities and differences, but summarize the results in two points. First, inclusion of age did not change the profile of z-scores for the terms also in the reference LMM (i.e., there is no difference in direction of effects between z-values in columns 1 and 3 and z-values are of very similar magnitude unless there is an interaction with age involved).

Second, as suggested by Electronic Supplementary Material Figure S1 and described above, there was a significant age x sex x secular-trend interaction for proxies of muscle power (b = -0.004, z = -3.09), in combination with a significant age x sex interaction (b = -0.23, z = -11.70). This constellation was also significant for relative muscle strength (b = -0.003, z = -2.31 for age x sex x secular trend; b = -0.07, z = -3.42 for age x sex). The strength panel in Electronic Supplementary Material Figure S1 suggests a larger sex effect and a steeper cubic trend for boys for the older than the younger children. Finally, sex effects were also estimated as significantly larger for older than younger children for cardiorespiratory endurance (b = -0.12, z = -3.25) and speed (b = -0.08, z = -3.98) – two effects that are also visible in Electronic Supplementary Material Figure S1. It is somewhat difficult to judge whether (some of) these age differences are genuine or possibly due to age differences in statistical power or test reliability for the current data.

In summary, there was exploratory evidence for *quantitative* *age differences* in our data, especially for proxies of muscle power and relative muscle strength. This difference should be followed up. We suspect that an important source of significant interactions with age is the different onset of puberty in boys and girls. We could not follow through with this hypothesis for three reasons. First, we did not have the relevant biological markers. Second, young children (especially boys) in the 1970s and old children in the final decade of the observation period were underrepresented in our data, but an analysis addressing this issue should include age as continuous variable and aim for a uniform age distribution across the observation period. Finally, even if the LMM including age was not degenerate, the ratio of number of observations (652) relative to number of model parameters (55) was marginal at best.

**Geographic/cultural region**

Do the results depend on the country or the geographic region of the systematic review? There were not enough studies from the same country to address the question at this level. There were three for Germany and Hungary and two from Australia, all other studies came from different countries. There was, however, a plausible geographic clustering of Australia and New Zealand (3 studies), Canada and the United States (2), Europe East (5), Europe North (3), Europe West (7), and cultural clustering of Brazil and Portugal (2). We added an additional random factor *World Region* comprising six levels to the reference LMM. This LMM converged without any numerical problems or degenerate estimates for variance components, but it did not improve the goodness of fit beyond the reference LMM; LRT-based χ^2^ (1) = 1.25, p = 0.26. Thus, we have no evidence for reliable differences between the regions of the world as described above. Electronic Supplementary Material Table S2 displays the fixed-effects estimates for both the world-region and the reference LMM to document that the profile of fixed effects was not affected by this model extension.

**Table S2** Fixed effects estimates of linear mixed model with world region as random factor

| **Component** | **Fixed-effect**  **estimates** | **z-values**  **world region** | **z-values**  **(ref LMM)** |
| --- | --- | --- | --- |
| **Cardiorespiratory endurance** | 0.32221 | **2.63** | **2.66** |
| Sex | 0.43665 | **8.12** | **8.23** |
| Year (linear) | -0.02402 | **-2.34** | **-2.36** |
| Year (quadratic) | -0.00365 | **-4.35** | **-4.35** |
| Year (cubic) | 0.00012 | **3.95** | **3.94** |
| Sex x year (linear) | -0.00791 | **-3.27** | **-3.28** |
| **Relative muscle strength** | -0.17348 | **-2.31** | **-2.40** |
| Sex | 0.08928 | 1.74 | 1.84 |
| Year (linear) | 0.01073 | 1.48 | 1.52 |
| Year (quadratic) | 0.00071 | **2.18** | **2.07** |
| Year (cubic) | -0.00004 | **-2.16** | **-2.14** |
| Sex x year (linear) | 0.00076 | 0.41 | 0.36 |
| **Proxies of muscle power** | 0.09128 | **2.43** | **2.79** |
| Sex | 0.35704 | **7.23** | **7.37** |
| Year (linear) | -0.00268 | -0.48 | -0.17 |
| Year (quadratic) | -0.00061 | -1.98 | **-2.04** |
| Year (cubic) | 0.00001 | 0.87 | 0.70 |
| Sex x year (linear) | 0.00479 | **2.85** | **2.81** |
| **Speed** | 0.02984 | 0.73 | 0.94 |
| Sex | 0.17867 | **3.53** | **3.67** |
| Year (linear) | -0.01971 | **-5.15** | **-4.90** |
| Year (quadratic) | -0.00040 | -1.87 | **-2.11** |
| Year (cubic) | 0.00006 | **4.62** | **4.60** |
| Sex x year (linear) | 0.00299 | 1.67 | 1.54 |

Note. The z-values in the right column are copied from Table 4 in our systematic review for ease of comparison between linear mixed model (LMM) with world region as random factor and the default LMMs reported here. Significant effects are in bold.

Of course, absence of evidence is not evidence of absence. The diversity of cultures represented by our studies is small compared to what is conceivable if we think about the entire world and a random factor with six levels would require very large cultural or geographic heterogeneity to yield a significant variance component.

**References**

1. Tomkinson GR, Lang JJ, Tremblay MS. Temporal trends in the cardiorespiratory fitness of children and adolescents representing 19 high-income and upper middle-income countries between 1981 and 2014. Br J Sports Med. 2019;53:478–86. doi:10.1136/bjsports-2017-097982.

**Electronic Supplementary Material Appendix S2. R script**

---

title: "An update on secular trends in physical fitness of children and adolescents from 1972 to 2015: A systematic review"

author: "Thea Fühner, Reinhold Kliegl, Fabian Arntz, Susi Kriemler, Urs Granacher"

date: "2020-08-08 (last revised: `r format(Sys.time())`)"

output:

html_document:

number_sections: yes

toc: yes

toc_depth: 2

toc_float: FALSE

code_folding: hide

df_print: paged

editor_options:

chunk_output_type: console

---

# Setup

```{r results='hide'}

options(warn=-1, dplyr.summarise.inform=FALSE) # Dangerous!

suppressPackageStartupMessages(library(readxl))

suppressPackageStartupMessages(library(splines))

suppressPackageStartupMessages(library(sjPlot))

suppressPackageStartupMessages(library(lme4))

suppressPackageStartupMessages(library(broom.mixed))

suppressPackageStartupMessages(library(remef))

suppressPackageStartupMessages(library(tidyverse))

suppressPackageStartupMessages(library(magrittr))

suppressPackageStartupMessages(library(modelr))

suppressPackageStartupMessages(library(cowplot))

# POOL SDs

poolSD <- function(sd, n)

# adapted from Gordon Smyth, 22 Jan 2004; https://rdrr.io/bioc/limma/src/R/poolvar.R

{

s2 <- as.vector(sd)^2

m <- 1/as.vector(n)

sm <- sum(m)

m <- m/sm

sqrt(sum(m * s2))

}

```

# Read data

The sheet `Data` in the Excel file contains the following variables:

+ Study - first author

+ pubYear - year of publication

+ age - age of children

+ Sex

+ Test

+ Component

+ year - year of assessment

+ N - n of children - extrapolated from total N for two studies (Pampakas, Sziva)

+ M - mean score

+ SD - standard deviation - computed from SE for Costa, Hardy, and Reiff

+ SE - standard error (when provided instead of SD)

Extrapolated Ns and SDs computed from SEs are marked in red in the excel spreadhsheet.

```{r}

data <- read_excel("SecularTrends_2020_08_13.xlsx", na="NA", sheet="data",

col_types = c("text", "numeric", "numeric", "text", "text", "text",

"numeric", "numeric", "numeric", "numeric", "numeric"))

data$Study <- factor(data$Study)

data$Sex <- factor(data$Sex)

data$Component <- factor(data$Component, levels=c("Endurance", "Strength", "Power", "Speed") )

dat <- data

# Quick check

dat %>% count(Study) %>% data.frame

```

# Contrasts and transformations

We center year of asssesment at 1990. Thus, the intercept estimates performance in this year. We compute indicator variables for linear quadratic, and cubic trends of `year` relative to this global reference.

```{r}

contrasts(dat$Component) <- contr.sum(4)

contrasts(dat$Sex) <- contr.sum(2)

dat$age_c <- dat$age - 12.5 # center at 12 years

dat$Age2 <- as_factor(if_else(dat$age <= 12.5, "6-12 years", "13-18 years"))

dat$Age2 <- factor(dat$Age2, levels=c("6-12 years", "13-18 years"))

# 1990 is global reference year (Grand Mean) for z

dat$year_c1 <- dat$year - 1990

dat$year_c2 <- dat$year_c1^2

dat$year_c3 <- dat$year_c1^3

dat %<>% relocate(age_c, Age2, .after=age)

dat %<>% relocate(year_c1:year_c3, .after=year)

# labels for studies

index <-

dat %>%

group_by(Component, Study) %>%

count() %>%

spread(Component, n, drop=FALSE) %>%

data.frame

```

# Compute z-scores (sample-weighted means and standard deviations)

+ Compute sample-weighted pooled mean `Mp`

+ Compute sample-weighted pooled standard deviation `SDp`

+ Compute `z`

```{r}

## Pool means and sd's of Study x Test x age cells

pool <- dat %>%

mutate(NM = N*M) %>%

group_by(age, Test, Study) %>%

summarise(Ns=sum(N), Mp = sum(NM)/sum(N),

SDp=poolSD(SD, N)) %>%

relocate(Study)

dat <- dat %>%

left_join(pool, by=c("Study", "age", "Test")) %>%

mutate(z = (M - Mp)/SDp) %>%

arrange(Study, age, Test)

# Invert time-based zs so that: positive values reflect positive change

timed_tests <- c("40 m sprint", "50 m sprint", "30 m sprint", "20 m sprint",

"10 x 5 m shuttle run", "1200 m run", "1600 m run", "3000 m run")

ix <- which(dat$Test %in% timed_tests)

dat[ix, "z"] <- (-1)*dat[ix, "z"]

```

# Linear mixed model (LMM)

## Model `LMM_z`

+ Model selection is documented in Supplementary Analysis 1.

+ Final model is refit here

+ Two variance components (`Endurance`, `Strength`) are supported by the data.

```{r fig.width = 10, fig.height = 4}

# generate indicator variables

mm_z <- model.matrix( ~ 0 + Component/(Sex*(year_c1 + year_c2 + year_c3)), dat)

Endurance <- mm_z[,1]

Strength <- mm_z[,2]

Power <- mm_z[,3]

Speed <- mm_z[,4]

attr(mm_z, "dimnames")[2][[1]][5:12]

# overall sex + linear trend for year

sex <- rowSums(mm_z[,5:8])

yl <- rowSums(mm_z[,9:12])

LMM_z <- lmer(z ~ 0 + Component/(Sex*year_c1 + year_c2 + year_c3) +

(0 + Endurance + Strength + sex +

mm_z[,9] + mm_z[,10] + mm_z[,11] || Study),

data=dat, REML=FALSE, control=lmerControl(calc.derivs=FALSE))

summary(rePCA(LMM_z))

VarCorr(LMM_z)

```

## Estimates of model parameters

```{r}

plbls_z <- c("Endurance", "Strength", "Power", "Speed",

rep(c("Sex"),4), rep(c("Year (lin)"),4), rep(c("Year (qdr)"),4),

rep(c("Year (cbc)"),4), rep(c("Sex x Year (lin)"),4))

fe_order <- c(1,5, 9,13,17,21, 2,6,10,14,18,22, 3,7,11,15,19,23, 4,8,12,16,20,24)

tab_model(LMM_z, pred.labels = plbls_z, digits=5, digits.re=5,

show.se=TRUE, show.stat=TRUE, show.ci=FALSE,

order.terms = fe_order)

for(i in 1:6) print(VarCorr(LMM_z)[[i]][1])

VarCorr(LMM_z)

```

## `z` Figure: `year` x `Sex` | `Component`

+ Generate `z` plot for cubic LMM specification.

```{r fig.width=6, fig.height=6}

# We drop two non-significant highest-order terms

names(fixef(LMM_z))

# Take out: Sex x year_c1 | Strength; year_c3 | Power; Sex x year_c1 | Speed

dat$pe_z <- keepef(LMM_z, fix=c(1:18, 20, 21, 23), ran=NULL)

# observed and partial-effect means

tab_z <- dat %>%

group_by(Component, Sex, year) %>%

summarise(N=n(), z=mean(z), pe_z=mean(pe_z))

# cubic Z function

p_Z <-

dat %>%

ggplot(aes(x=year, y=pe_z, group=Sex, linetype=Sex, shape=Sex)) +

geom_hline(yintercept=0) + geom_vline(xintercept=1990) +

geom_point(data=tab_z, aes(y=z)) +

geom_smooth(method="lm", formula=y ~ poly(x,3, raw=TRUE), se=TRUE, color="black") +

facet_grid(. ~ Component) +

scale_x_continuous("", breaks=seq(1974, 2014, 8), limits = c(1972,2015)) +

scale_y_continuous("z-score", breaks=seq(-2, +2, .5)) +

coord_cartesian(ylim=c(-2,+2)) + theme_bw() +

theme(legend.position="none")

```

## Empirical approximation and plot of first derivative

+ Generate predictions for partial effects

+ Compute first derivative (empirical)

+ Generate `Z` + `dZ` plot

```{r fig.width=10, fig.heigth=8}

# function for LMM partial effects

lm_y3 <- function(df) lm(pe_z ~ 1 + year_c1 + year_c2 + year_c3, data = df)

# generate predictions in `pred` within Sex x Component

pred_y3 <-

dat %>%

group_by(Sex, Component) %>%

nest() %>%

mutate(model = map(data, lm_y3),

fitted = map2(data, model, add_predictions))

# compute first derivative

df <-

unnest(pred_y3, fitted) %>%

group_by(Sex, Component, year) %>%

summarise(N=n(), Z=mean(pred)) %>%

mutate(dZ = (lead(Z)-Z)/(lead(year)-year)) %>%

filter(!is.na(dZ))

# generate dZ plot

p_dZ <- df %>%

ggplot(aes(x=year, y=dZ, group=Sex, linetype=Sex, shape=Sex)) +

geom_smooth(method ="lm", formula = y ~ poly(x,3, raw=TRUE), se=FALSE, color="black") +

facet_grid(. ~ Component) +

scale_x_continuous("", breaks=seq(1974, 2014, 8), limits = c(1972,2015)) +

scale_y_continuous("delta z-score", breaks=seq(-0.1, +0.1, .05)) +

geom_hline(yintercept=0) + geom_vline(xintercept=1990) +

coord_cartesian(ylim=c(-0.13, +0.13)) +

theme_bw() +

theme(legend.position=c(.99, .01), legend.justification=c(.99,.01))

# Combine `Z` and `dZ` component plots

plot_grid(p_Z, p_dZ, labels = c('a', 'b'), nrow=2, label_size = 12, align="v")

ggsave("figures/F2_Year_Sex_Comp_for_Z_dZ.pdf", width = 10, height = 8)

```

## Study-level conditional means

Generate plot of `LMM_z` conditional means for variance components `Endurance` and `Strength` supported by data.

```{r fig.width=10, fig.height=8}

CondMeans <- as.data.frame(ranef(LMM_z, condVar=TRUE))

CondMeans[CondMeans$condval == 0, c("condval", "condsd") ] <- NA

levels(CondMeans$term)[3:6] <- c("Sex", "Year[Endurance]", "Year[Strength]", "Year[Power]")

ggplot(CondMeans, aes(y=grp, x=condval)) +

geom_point() + facet_wrap(~term,scales="free_x") +

geom_errorbarh(aes(xmin = condval -2*condsd,

xmax = condval +2*condsd), height=0) +

geom_vline(xintercept=0) + xlab("Conditional Means") +

ylab("") + theme_bw()

ggsave("figures/F3_CondMeans.pdf", width=10, height=8)

```

There is larger between-study heterogeneity for `Endurance` than `Strength` and no support for between-study heterogeneity for `Power` and `Speed`. There is also heterogeneity between studies relating to the `Sex` effect and linear slopes of `year_c1` for `Endurance`, `Strength` and `Power`.

## Observation-level residuals

```{r fig.width=10, fig.height=8}

aug_LMM_z <- augment(LMM_z)

aug_LMM_z <-

tibble(cbind(aug_LMM_z, year=dat$year)) %>%

relocate(year, .after=Sex)

p1_r <-

aug_LMM_z %>%

ggplot(aes(year, .resid)) +

geom_point(colour="black", size=1) +

facet_grid(.~Component) +

geom_hline(yintercept=0) +

geom_smooth(method="lm", color="black") +

ylab("LMM residual") + xlab("") +theme_bw() +

coord_cartesian(ylim=c(-1,+1))

p2_r <-

aug_LMM_z %>%

ggplot(aes(sample=.resid/sd(.resid))) + ## scale to variance=1

stat_qq(aes(group=1)) +

xlab("Standard normal quantile") +

ylab("Standardized LMM residual") +

geom_abline(intercept=0, slope=1) +

coord_fixed(ratio=1) + theme_bw()

p3_r <-

aug_LMM_z %>%

ggplot(aes(.fitted, .resid)) +

geom_point(colour="black", size=1) +

xlab("Fitted LMM value") + ylab("LMM residual") +

geom_hline(yintercept=0) + # geom_density2d(size=1) + # + geom_hex()

theme_bw() +

coord_cartesian(ylim=c(-1,+1))

first_row = plot_grid(p1_r, labels = c('a'))

second_row = plot_grid(p2_r, p3_r, labels = c('b', 'c'), nrow = 1)

plot_grid(first_row, second_row, labels=c('', ''), ncol=1)

ggsave("figures/F4_LMM_Resid.pdf", width = 10, height = 8)

```

# Supplementary control analyses

## Model selection

1. Check VCs for `Component`

2. Check VCs for `Sex`, `year_c1`, `year_c2`, and `year_c3`

3. Check correlation parameters for VCs supported by data.

### VCs for `Component`

```{r}

# STAGE 1

# fit LMM with 4 test components

LMM_z_4C <- lmer(z ~ 0 + Component/(Sex*(year_c1 + year_c2 + year_c3)) +

(0 + Endurance + Strength + Power + Speed || Study),

data=dat, REML=FALSE, control=lmerControl(calc.derivs=FALSE))

summary(rePCA(LMM_z_4C)) # overparameterized

VarCorr(LMM_z_4C)

# w/o speed

LMM_z_nospd <- lmer(z ~ 0 + Component/(Sex*(year_c1 + year_c2 + year_c3)) +

(0 + Endurance + Strength + Power || Study),

data=dat, REML=FALSE, control=lmerControl(calc.derivs=FALSE))

anova(LMM_z_nospd, LMM_z_4C) # not significant

# w/o power

LMM_z_nopow <- lmer(z ~ 0 + Component/(Sex*(year_c1 + year_c2 + year_c3)) +

(0 + Endurance + Strength + Speed || Study),

data=dat, REML=FALSE, control=lmerControl(calc.derivs=FALSE))

anova(LMM_z_nopow, LMM_z_4C) # not significant

# w/o strength

LMM_z_nostr <- lmer(z ~ 0 + Component/(Sex*(year_c1 + year_c2 + year_c3)) +

(0 + Endurance + Power + Speed || Study),

data=dat, REML=FALSE, control=lmerControl(calc.derivs=FALSE))

anova(LMM_z_nostr, LMM_z_4C) # not significant

# w/o endurance

LMM_z_noend <- lmer(z ~ 0 + Component/(Sex*(year_c1 + year_c2 + year_c3)) +

(0 + Strength + Power + Speed || Study),

data=dat, REML=FALSE, control=lmerControl(calc.derivs=FALSE))

anova(LMM_z_noend, LMM_z_4C) # significant!

# Therefore,

# only endurance + strength

LMM_z_end_str <- lmer(z ~ 0 + Component/(Sex*(year_c1 + year_c2 + year_c3)) +

(0 + Endurance + Strength || Study),

data=dat, REML=FALSE, control=lmerControl(calc.derivs=FALSE))

anova(LMM_z_end_str, LMM_z_4C)

```

Variance components `Endurance` and `Strength` supported by data; `Strength` and `Power` were not.

We use this LMM in the first version.

```{r eval=FALSE}

LMM_z <- LMM_z_end_str

```

### VC for `sex`

Must use indicator variable for `sex` in RE part.

```{r}

attr(mm_z, "dimnames")[2][[1]][5:8]

# overall

sex <- rowSums(mm_z[,5:8])

LMM_z_sex <- lmer(z ~ 0 + Component/(Sex*(year_c1 + year_c2 + year_c3)) +

(0 + Endurance + Strength + sex || Study),

data=dat, REML=FALSE, control=lmerControl(calc.derivs=FALSE))

VarCorr(LMM_z_sex)

anova(LMM_z, LMM_z_sex)

# component_wise

LMM_z_sex4 <- lmer(z ~ 0 + Component/(Sex*(year_c1 + year_c2 + year_c3)) +

(0 + Endurance + Strength +

mm_z[,5] + mm_z[,6] + mm_z[,7] + mm_z[,8] || Study),

data=dat, REML=FALSE, control=lmerControl(calc.derivs=FALSE))

VarCorr(LMM_z_sex4)

anova(LMM_z, LMM_z_sex, LMM_z_sex4)

```

+ `Sex` effect varies significantly between studies

+ No improvement in LRT for component-wise specification

### VCs for `sex` + `year_c1`

```{r}

attr(mm_z, "dimnames")[2][[1]][5:12]

# overall sex + linear trend for year

sex <- rowSums(mm_z[,5:8])

yl <- rowSums(mm_z[,9:12])

LMM_z_sex_yl <- lmer(z ~ 0 + Component/(Sex*(year_c1 + year_c2 + year_c3)) +

(0 + Endurance + Strength + sex + yl || Study),

data=dat, REML=FALSE, control=lmerControl(calc.derivs=FALSE))

VarCorr(LMM_z_sex_yl)

anova(LMM_z, LMM_z_sex, LMM_z_sex_yl)

# component_wise

LMM_z_sex_yl4 <- lmer(z ~ 0 + Component/(Sex*(year_c1 + year_c2 + year_c3)) +

(0 + Endurance + Strength + sex +

mm_z[,9] + mm_z[,10] + mm_z[,11] + mm_z[,12] || Study),

data=dat, REML=FALSE, control=lmerControl(calc.derivs=FALSE))

summary(rePCA(LMM_z_sex_yl4))

VarCorr(LMM_z_sex_yl4)

LMM_z_sex_yl3 <- lmer(z ~ 0 + Component/(Sex*(year_c1 + year_c2 + year_c3)) +

(0 + Endurance + Strength + sex +

mm_z[,9] + mm_z[,10] + mm_z[,11] || Study),

data=dat, REML=FALSE, control=lmerControl(calc.derivs=FALSE))

summary(rePCA(LMM_z_sex_yl3))

VarCorr(LMM_z_sex_yl3)

anova(LMM_z, LMM_z_sex, LMM_z_sex_yl, LMM_z_sex_yl3, LMM_z_sex_yl4)

```

+ `sex` (overall) + `year_c1` (component-wise) effects vary significantly between studies

+ VC for lin effect within `Speed` can be dropped (Chi-sq = 3.2, p=0.073).

### VCs for `sex` + `year_c1` + `year_c2`

We do not expect further improvement because there were only a few studies with more than two assessments.

```{r eval=FALSE}

attr(mm_z, "dimnames")[2][[1]][9:16]

# overall sex + linear + quadratic trendsfor year

sex <- rowSums(mm_z[,5:8])

yl <- rowSums(mm_z[,9:12])

yq <- rowSums(mm_z[,13:16])

LMM_z_syl4q <- lmer(z ~ 0 + Component/(Sex*(year_c1 + year_c2 + year_c3)) +

(0 + Endurance + Strength + sex +

mm_z[,9] + mm_z[,10] + mm_z[,11] + mm_z[,12] + yq || Study),

data=dat, REML=FALSE, control=lmerControl(calc.derivs=FALSE))

summary(rePCA(LMM_z_syl4q)) # overparameterized

VarCorr(LMM_z_syl4q)

# ... just to make sure

LMM_z_syl4q4 <- lmer(z ~ 0 + Component/(Sex*(year_c1 + year_c2 + year_c3)) +

(0 + Endurance + Strength + sex + yl +

mm_z[, 9] + mm_z[,10] + mm_z[,11] + mm_z[,12] +

mm_z[,13] + mm_z[,14] + mm_z[,15] + mm_z[,16] || Study),

data=dat, REML=FALSE, control=lmerControl(calc.derivs=FALSE))

summary(rePCA(LMM_z_syl4q4))

VarCorr(LMM_z_syl4q4)

anova(LMM_z_sex, LMM_z_sex_yl, LMM_z_sex_yl4, LMM_z_syl4q, LMM_z_syl4q4)

```

No further improvement.

### VCs for `sex` + `year_c1` + `year_c2` + `year_c3`

We do not expect further improvement because there were only a few studies with more than three assessments.

```{r eval=FALSE}

attr(mm_z, "dimnames")[2][[1]][5:20]

# overall sex + linear + qudaratic + cubic trend for year

sex <- rowSums(mm_z[,5:8])

yl <- rowSums(mm_z[ ,9:12])

yq <- rowSums(mm_z[,13:16])

yc <- rowSums(mm_z[,17:20])

LMM_z_syl4qc <- lmer(z ~ 0 + Component/(Sex*(year_c1 + year_c2 + year_c3)) +

(0 + Endurance + Strength + sex +

mm_z[,9] + mm_z[,10] + mm_z[,11] + mm_z[,12] +

yq + yc || Study),

data=dat, REML=FALSE, control=lmerControl(calc.derivs=FALSE))

summary(rePCA(LMM_z_syl4qc)) # overparameterized

VarCorr(LMM_z_syl4qc)

# ... just make sure

LMM_z_syl4qc4 <- lmer(z ~ 0 + Component/(Sex*(year_c1 + year_c2 + year_c3)) +

(0 + Endurance + Strength + sex + yl +

mm_z[, 9] + mm_z[,10] + mm_z[,11] + mm_z[,12] + yq +

mm_z[,17] + mm_z[,18] + mm_z[,19] + mm_z[,20] || Study),

data=dat, REML=FALSE, control=lmerControl(calc.derivs=FALSE))

summary(rePCA(LMM_z_syl4q4)) # overparameterized

VarCorr(LMM_z_syl4q4)

anova(LMM_z_sex, LMM_z_sex_yl, LMM_z_sex_yl4, LMM_z_syl4q, LMM_z_syl4qc, LMM_z_syl4qc4)

```

No further improvement.

### Correlation parameters

```{r eval=FALSE}

LMM_z_sex_yl3_cp_full <- lmer(z ~ 0 + Component/(Sex*(year_c1 + year_c2 + year_c3)) +

(0 + Endurance + Strength +

sex + mm_z[, 9] + mm_z[,10] + mm_z[,11] | Study),

data=dat, REML=FALSE, control=lmerControl(calc.derivs=FALSE))

summary(rePCA(LMM_z_sex_yl3_cp_full)) # overparameterized

VarCorr(LMM_z_sex_yl3_cp_full)

anova(LMM_z_sex_yl3, LMM_z_sex_yl3_cp_full) # marginally significant

# reducing CPs, keeping sex and yl for endurance

LMM_z_sex_yl3_cp1 <- lmer(z ~ 0 + Component/(Sex*(year_c1 + year_c2 + year_c3)) +

(0 + Strength + sex + mm_z[, 10] + mm_z[,11] || Study) +

(0 + Endurance + mm_z[,9] | Study),

data=dat, REML=FALSE, control=lmerControl(calc.derivs=FALSE))

summary(rePCA(LMM_z_sex_yl3_cp1))

VarCorr(LMM_z_sex_yl3_cp1)

anova(LMM_z_sex_yl3, LMM_z_sex_yl3_cp1, LMM_z_sex_yl3_cp_full)

# Alternative

LMM_z_sex_yl3_cp2 <- lmer(z ~ 0 + Component/(Sex*(year_c1 + year_c2 + year_c3)) +

(0 + sex + mm_z[,9] | Study) +

(0 + Endurance + Strength + mm_z[, 10] + mm_z[,11] || Study),

data=dat, REML=FALSE, control=lmerControl(calc.derivs=FALSE))

summary(rePCA(LMM_z_sex_yl3_cp2))

VarCorr(LMM_z_sex_yl3_cp2)

anova(LMM_z_sex_yl3, LMM_z_sex_yl3_cp2, LMM_z_sex_yl3_cp_full) # not significant

```

There are various specifications that work, but not together

+ CP of `sex` and `year_c1` for `Endurance` is supported by data (r=-0.467). Studies with a large effect of sex (boys - girls) decline less across years in the `Endurance` component.

+ CP of `Endurance` and `year_c1` for `Endurance` is supported by data (r=-0.516). Studies with a large edurance effect decline less across years in the `Endurance` component.

The CPs are not significant individually and also not when entered together.

### Final selection

The selection procedure suggests that for the random factor `Study`

+ reliable VCs for `Endurance` and `Strength`

+ reliable VC for `Sex`

+ reliable VCs for `year_c1` for `Endurance`, `Strength`, and `Power`

+ questionable negative correlation parameter `Endurance` and `year_c1 | Endurance` or

questionable negative correlation parameter `Sex` and `year_c1 | Endurance`

+ no reliable VCs for quadratic and cubic yearly trends

We use the VC-only LMM in the second version and check the higher-order interactions.

```{r}

LMM_z3 <- LMM_z_sex_yl3

LMM_z2 <- lmer(z ~ 0 + Component/(Sex*(year_c1 + year_c2) + year_c3) +

(0 + Endurance + Strength + sex +

mm_z[,9] + mm_z[,10] + mm_z[,11] || Study),

data=dat, REML=FALSE, control=lmerControl(calc.derivs=FALSE))

summary(rePCA(LMM_z2))

VarCorr(LMM_z2)

anova(LMM_z2, LMM_z3)

LMM_z1 <- lmer(z ~ 0 + Component/(Sex*year_c1 + year_c2 + year_c3) +

(0 + Endurance + Strength + sex +

mm_z[,9] + mm_z[,10] + mm_z[,11] || Study),

data=dat, REML=FALSE, control=lmerControl(calc.derivs=FALSE))

summary(rePCA(LMM_z1))

VarCorr(LMM_z1)

anova(LMM_z1, LMM_z2, LMM_z3)

LMM_z <- LMM_z1

```

## Adding `Age2` as covariate

+ Faceting on `Age2` with `LMM_z` partial effects

+ Adding `Age2` as covariate

+ Figure 2A with new fit

```{r}

p_Z_Age2 <-

dat %>%

ggplot(aes(x=year, y=pe_z, group=Sex, linetype=Sex, shape=Sex)) +

geom_hline(yintercept=0) + geom_vline(xintercept=1990) +

geom_point(data=tab_z, aes(y=pe_z)) +

geom_smooth(method="lm", formula=y ~ poly(x,3, raw=TRUE), se=TRUE, color="black") +

facet_grid(Age2 ~ Component) +

scale_x_continuous("", breaks=seq(1974, 2014, 8), limits = c(1972,2015)) +

scale_y_continuous("z-score", breaks=seq(-2, +2, .5)) +

coord_cartesian(ylim=c(-2,+2)) + theme_bw() +

theme(legend.position=c(.99, .01), legend.justification=c(.99,.01))

p_Z_Age2

ggsave("figures/F5_Year_Sex_Comp_for_Z_Age2.pdf", width = 10, height = 8)

contrasts(dat$Age2) <- contr.sum(2)

LMM_z_Age2_1 <- lmer(z ~ 0 + Component/((Sex*year_c1 + year_c2 + year_c3) + Age2) +

(0 + Endurance + Strength + sex +

mm_z[,9] + mm_z[,10] + mm_z[,11] || Study),

data=dat, REML=FALSE, control=lmerControl(calc.derivs=FALSE))

summary(rePCA(LMM_z_Age2_1))

VarCorr(LMM_z_Age2_1)

anova(LMM_z, LMM_z_Age2_1)

print(summary(LMM_z_Age2_1))

LMM_z_Age2 <- lmer(z ~ 0 + Component/((Sex*year_c1 + year_c2 + year_c3)*Age2) +

(0 + Endurance + Strength + sex +

mm_z[,9] + mm_z[,10] + mm_z[,11] || Study),

data=dat, REML=FALSE, control=lmerControl(calc.derivs=FALSE))

summary(rePCA(LMM_z_Age2))

VarCorr(LMM_z_Age2)

anova(LMM_z, LMM_z_Age2)

tab_model(LMM_z_Age2, digits=5, digits.re=5, show.se=TRUE, show.stat=TRUE, show.ci=FALSE)

```

+ `LMM_z` predictions recover secular trends for young and old children (i.e., without refit of LMM)

+ ...

## World region

```{r}

wr <- read_excel("SecularTrends_2020_08_13.xlsx", na="NA", sheet="wrld_rgn",

col_types = c("text", "text", "text"))

wr$Country <- factor(wr$Country)

wr$WorldRegion <- factor(wr$WorldRegion)

dat <- dat %>% left_join(wr, by="Study")

LMM_z_wr <- update(LMM_z, . ~ . + (1 | WorldRegion), data=dat)

summary(rePCA(LMM_z_wr))

VarCorr(LMM_z)

VarCorr(LMM_z_wr)

anova(LMM_z, LMM_z_wr)

plbls_z <- c("Endurance", "Strength", "Power", "Speed",

rep(c("Sex"),4), rep(c("Year (lin)"),4), rep(c("Year (qdr)"),4),

rep(c("Year (cbc)"),4), rep(c("Sex x Year (lin)"),4))

tab_model(LMM_z_wr,

pred.labels = plbls_z, digits=5, digits.re=5,

show.se=FALSE, show.stat=TRUE, show.ci=FALSE,

order.terms = c(1,5, 9,13,17,21, 2,6,10,14,18,22,

3,7,11,15,19,23, 4,8,12,16,20,24))

VarCorr(LMM_z_wr)

VarCorr(LMM_z)

```

# R version and packages

```{r}

sessionInfo()

```

```

## R version 4.0.2 (2020-06-22)

## Platform: x86_64-apple-darwin17.0 (64-bit)

## Running under: macOS Catalina 10.15.6

##

## Matrix products: default

## BLAS: /Library/Frameworks/R.framework/Versions/4.0/Resources/lib/libRblas.dylib

## LAPACK: /Library/Frameworks/R.framework/Versions/4.0/Resources/lib/libRlapack.dylib

##

## locale:

## [1] en_US.UTF-8/en_US.UTF-8/en_US.UTF-8/C/en_US.UTF-8/en_US.UTF-8

##

## attached base packages:

## [1] splines stats graphics grDevices utils datasets methods

## [8] base

##

## other attached packages:

## [1] cowplot_1.0.0 modelr_0.1.8 magrittr_1.5 forcats_0.5.0

## [5] stringr_1.4.0 dplyr_1.0.2 purrr_0.3.4 readr_1.3.1

## [9] tidyr_1.1.2 tibble_3.0.3 ggplot2_3.3.2 tidyverse_1.3.0

## [13] remef_1.0.7 broom.mixed_0.2.6 lme4_1.1-23.9000 Matrix_1.2-18

## [17] sjPlot_2.8.4 readxl_1.3.1

##

## loaded via a namespace (and not attached):

## [1] nlme_3.1-149 fs_1.5.0 lubridate_1.7.9 insight_0.9.1

## [5] httr_1.4.2 tools_4.0.2 TMB_1.7.18 backports_1.1.9

## [9] R6_2.4.1 sjlabelled_1.1.6 mgcv_1.8-33 DBI_1.1.0

## [13] colorspace_1.4-1 withr_2.2.0 tidyselect_1.1.0 emmeans_1.5.0

## [17] compiler_4.0.2 performance_0.4.8 cli_2.0.2 rvest_0.3.6

## [21] xml2_1.3.2 sandwich_2.5-1 labeling_0.3 bayestestR_0.7.2

## [25] scales_1.1.1 mvtnorm_1.1-1 digest_0.6.25 minqa_1.2.4

## [29] rmarkdown_2.3 pkgconfig_2.0.3 htmltools_0.5.0 dbplyr_1.4.4

## [33] rlang_0.4.7 rstudioapi_0.11 farver_2.0.3 generics_0.0.2

## [37] zoo_1.8-8 jsonlite_1.7.0 parameters_0.8.2 Rcpp_1.0.5

## [41] munsell_0.5.0 fansi_0.4.1 lifecycle_0.2.0 stringi_1.4.6

## [45] multcomp_1.4-13 yaml_2.2.1 snakecase_0.11.0 MASS_7.3-52

## [49] plyr_1.8.6 grid_4.0.2 blob_1.2.1 sjmisc_2.8.5

## [53] crayon_1.3.4 lattice_0.20-41 ggeffects_0.15.1 haven_2.3.1

## [57] sjstats_0.18.0 hms_0.5.3 knitr_1.29 pillar_1.4.6

## [61] boot_1.3-25 estimability_1.3 effectsize_0.3.2 reshape2_1.4.4

## [65] codetools_0.2-16 reprex_0.3.0 glue_1.4.2 evaluate_0.14

## [69] vctrs_0.3.4 nloptr_1.2.2.2 cellranger_1.1.0 gtable_0.3.0

## [73] assertthat_0.2.1 xfun_0.16 xtable_1.8-4 broom_0.7.0.9000

## [77] coda_0.19-3 survival_3.2-3 statmod_1.4.34 TH.data_1.0-10

## [81] ellipsis_0.3.1

```

**Compliance with Ethical Standards**

**Funding source**

No sources of funding were used to assist in the preparation on this article.

**Conflicts of interest/Competing interests**

Thea Fühner, Reinhold Kliegl, Fabian Arntz, Susi Kriemler, and Urs Granacher declare that they have no conflicts of interest relevant to the content of this systematic review.

**Availability of data and material**

R script is available in Electronic Supplementary Material Appendix S2. Data from the included studies are available in Electronic Supplementary Material Appendix S3. Furthermore, R script and data from included studies are available at the Open Science Framework: https://osf.io/rs7x2/?view_only=090b2e63c23e4e719f766f6791ed1a89

**Code availability**

Not applicable

**Authors’ contributions**

TF, RK, SK, and UG: made substantial contributions to conception and design; TF and FA: contributed to data collection; RK and TF carried out data analysis; TF, RK, SK, and UG: interpreted the data; TF: wrote the first draft of the manuscript and all authors were involved in revising it critically for important intellectual content; all authors provide final approval of the version to be published and agreed to be accountable for all aspects of the work.
